# Biophysical properties of Na_V_1.5 channels from atrial-like and ventricular-like cardiomyocytes derived from human induced pluripotent stem cells

**DOI:** 10.1038/s41598-023-47310-6

**Published:** 2023-11-24

**Authors:** Charles-Albert Chapotte-Baldacci, Marion Pierre, Mohammed Djemai, Valérie Pouliot, Mohamed Chahine

**Affiliations:** 1 Department of Medicine, Laval University, Quebec City, QC Canada; 2 CERVO Brain Research Centre, 2601, chemin de la Canardière, Quebec City, QC G1J 2G3 Canada

**Keywords:** Biological techniques, Biophysics, Cell biology, Stem cells, Cardiology

## Abstract

Generating atrial-like cardiomyocytes derived from human induced pluripotent stem cells (hiPSCs) is crucial for modeling and treating atrial-related diseases, such as atrial arrythmias including atrial fibrillations. However, it is essential to obtain a comprehensive understanding of the electrophysiological properties of these cells. The objective of the present study was to investigate the molecular, electrical, and biophysical properties of several ion channels, especially Na_V_1.5 channels, in atrial hiPSC cardiomyocytes. Atrial cardiomyocytes were obtained by the differentiation of hiPSCs treated with retinoic acid (RA). The quality of the atrial specification was assessed by qPCR, immunocytofluorescence, and western blotting. The electrophysiological properties of action potentials (APs), Ca^2+^ dynamics, K^+^ and Na^+^ currents were investigated using patch-clamp and optical mapping approaches. We evaluated mRNA transcript and protein expressions to show that atrial cardiomyocytes expressed higher atrial- and sinoatrial-specific markers (*MYL7*, *CACNA1D*) and lower ventricular-specific markers (*MYL2*, *CACNA1C*, *GJA1*) than ventricular cardiomyocytes. The amplitude, duration, and steady-state phase of APs in atrial cardiomyocytes decreased, and had a shape similar to that of mature atrial cardiomyocytes. Interestingly, Na_V_1.5 channels in atrial cardiomyocytes exhibited lower mRNA transcripts and protein expression, which could explain the lower current densities recorded by patch-clamp. Moreover, Na^+^ currents exhibited differences in activation and inactivation parameters. These differences could be explained by an increase in *SCN2B* regulatory subunit expression and a decrease in *SCN1B* and *SCN4B* regulatory subunit expressions. Our results show that a RA treatment made it possible to obtain atrial cardiomyocytes and investigate differences in Na_V_1.5 channel properties between ventricular- and atrial-like cells.

## Introduction

The voltage-gated sodium channel (VGSC) α subunit, Na_V_1.5, which is encoded by the *SCN5A* gene, is the predominant Na^+^ channel in the heart. Na_V_1.5 plays a vital role in the generation and propagation of electrical impulses throughout the heart^1^, and its activation contributes to the rising phase (phase 0) of cardiac action potentials (APs). In the mammalian heart, AP shape and duration vary depending on the regions and chambers (atrial *vs.* ventricular)^2^. Marked differences in the biophysical properties of Na_V_1.5 have been described in atrial and ventricular cardiomyocytes from several species^2–4^. These differences are closely related to the expression of regulatory β-subunits, which act as regulators of the cell surface expression and gating properties of VGSCs^5^. The evidence for chamber-specific differences in Na^+^ currents in the human heart have been also reported^6,7^.Many *SCN5A* variants have been reported to cause channelopathies that are responsible for cardiac arrhythmia and sudden death, including long QT syndrome^8,9^, Brugada syndrome^10^, conduction abnormalities^11^, atrial fibrillation^12^, and dilated cardiomyopathy^13,14^.

The generation of cardiomyocytes derived from human induced pluripotent stem cells (hiPSC-CMs) has been predominantly used to model inherited cardiac diseases that mainly affect ventricular cardiomyocytes^15^. Recently, these cells have been successfully differentiated into atrial cardiomyocytes^16^, opening the way for modeling atrial-specific inherited arrhythmia. More specifically, atrial-like hiPSC-CMs have been employed to model genetic manifestations of atrial fibrillation, either by directly utilizing patient samples^17^ or through the integration of *SCN5A* variants using the CRISPR-Cas9 strategy^18^. Nevertheless, the electrophysiological traits of healthy atrial-like hiPSC-CMs remain inadequately characterized and comprehended.

The purpose of the present study was to conduct a comprehensive investigation and comparison of the biophysical characteristics of various ion channels, with a particular focus on Na_V_1.5 channels and their associated regulatory subunits. We aimed to explore these properties in two distinct types of cardiomyocytes: control ventricular cardiomyocytes (vCMs) and cardiomyocytes stimulated by retinoic acid (aCMs). We confirmed the atrial molecular specification mediated by the retinoic acid treatment, using several ventricular-, atrial-, and sinoatrial-specific markers. Notably, our study revealed substantial electrophysiological disparities between vCMs and aCMs, both at the level of single-cells and within cell monolayers. Of particular interest, we observed that Na_V_1.5 channels were not equally expressed in vCMs and aCMs, and the Na^+^ currents exhibited marked differences in both their densities and biophysical properties.

## Results

### Characterization of the atrial specification of hiPSC-CMs

hiPSCs have been widely used in biomedical research for over 10 years to obtain large quantities of cells that are able to reproduce human physiology and pathologies. Several differentiation protocols have been developed to obtain hiPSC-derived cardiomyocytes with a mainly ventricular phenotype. In recent years, retinoic acid has been commonly used to drive the atrial specification of hiPSC-CMs^19–21^.

**Figure 1:**
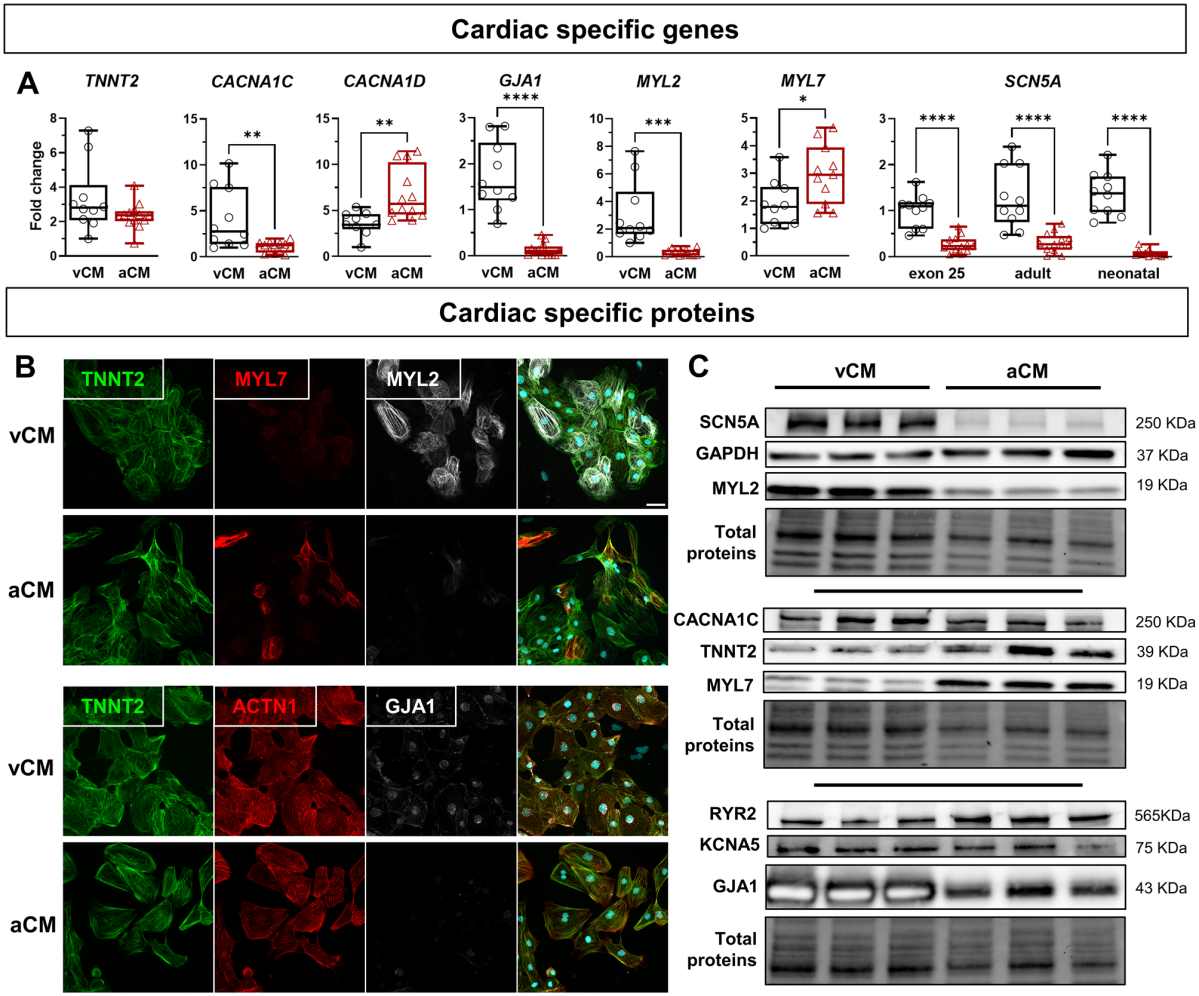
Characterization of specific-markers of vCMs and aCMs. (**A**) qPCR analysis of several cardiomyocyte genes implicated in cellular excitability (*SCN5A, CACNA1C, CACNA1D, GJA1*) and contraction (*TNNT2, MYL2, MYL7*). The analysis of exon 25 of *SCN5A* mRNA covered all isoforms, including the adult and neonatal isoforms. (**B**) Fluorescence images showing immunolabeling of cardiac TNNT2, MYL7, MYL2, ACTN1 (α-actinin), GJA1 (connexin 43), and nuclei (DAPI, cyan) (scale bar: 40 µm). Immunofluorescence images were acquired using Zeiss LSM780 confocal microscope, processed with ZEN software (Zeiss), and adapted with ImageJ software version 1.54f (NIH, Bethesda, MD, USA). (**C**) Western blot analysis of the expression of several excitation–contraction coupling proteins and ion channels in vCMs and aCMs. All images of cropped blot section were exposed with an optimal time to observe protein bands. All cropped blot sections were delimited by black lines. Cropped strain-free blots showing total proteins served as loading control. Original blots are presented in Suppl. Fig. S4. Top panel, middle panel, and bottom panel, respectively, refer to “Blot 1”, Blot 2” and “Blot 3″ in Suppl. Fig. S4. Cropped section areas are indicated in Suppl. Fig. S4 by red lines. Immunoblot images were adapted with ImageJ software and arranged with Microsoft Powerpoint software version microsoft 365 (Microsoft, Redmond, WA, USA).

To initiate the atrial specification of hiPSC-CMs, cells were differentiated using a commercial protocol and were treated with 1 µmol/L RA from D2 to D5. Atrial specification was first evaluated by qPCR, immunocytofluorescence, and western blotting. Higher expressions of ventricular-specific markers *MYL2* and *GJA1* (Fig. 1A–C; Suppl. Fig. S1A) were observed in ventricular-like cardiomyocytes derived from hiPSCs (vCMs). In comparison, higher expressions of atrial- and sinoatrial-specific markers *MYL7* (Fig. 1A–C; Suppl. Fig. S1A) and *CACNA1D* (Fig. 1A) were detected in atrial-like cardiomyocytes derived from hiPSCs (aCMs). In vCMs, a significant 3.4-fold increase in *MYL2* gene expression was observed compared to *MYL7* (Suppl. Fig. S2A). Conversely, in aCMs, a significant shift occurred, with *MYL7* gene expression being 2.8-fold higher than *MYL2* (Suppl. Fig. S2B). The immunostaining of GJA1 indicated a perinuclear pattern found in vCMs and aCMs (Fig. 1B; Suppl. Fig. S1B) and a punctuated pattern located at the plasma membrane in vCMs that was not detected in aCMs (Fig. 1B). We also observed no change in the cardiac marker *TNNT2* transcript expression in both cell types but a higher TNNT2 protein level in aCMs (Fig. 1A,C; Suppl. Fig. S1A). In addition, the calcium release mediator RYR2 is expressed at a higher level in aCMs (Fig. 1C; Suppl. Fig. S1A), which could reflect an improved differentiation of aCMs. Nevertheless, a decrease of *CACNA1C* transcript level was detected in aCMs (Fig. 1A), but this was not confirmed at the protein level where no change was observed (Fig. 1C; Suppl. Fig. S1A). No significant change has been measured in KCNA5 channel expression between both cell types (Fig. 1C; Suppl. Fig. S1A). Furthermore, the *CACNA1C* gene expression was significatively higher than *CACNA1D* in vCMs (168-fold higher) (Suppl. Fig. S2C) and aCMs (4.6-fold higher) (Suppl. Fig. S2D), indicating that it mainly contributed to L-type Ca^2+^ currents. Surprisingly, there was a marked reduction in the expression of the Na_V_1.5 channel in aCMs compared to vCMs, as quantified at the mRNA level by a decrease in the expression of all *SCN5A* isoforms (neonatal and adult) (Fig. 1A) and, as well as at the protein level (Fig. 1C; Suppl. Fig. S1A). Furthermore, the neonatal isoform of *SCN5A* was significantly more expressed in both cell types compared to the adult isoform (Suppl. Fig. S3A,B), but we observed a small increase of the adult isoform of *SCN5A* in aCMs (Suppl. Fig. S3C), which is often associated with an increase in cardiomyocyte maturity.

**Figure 2:**
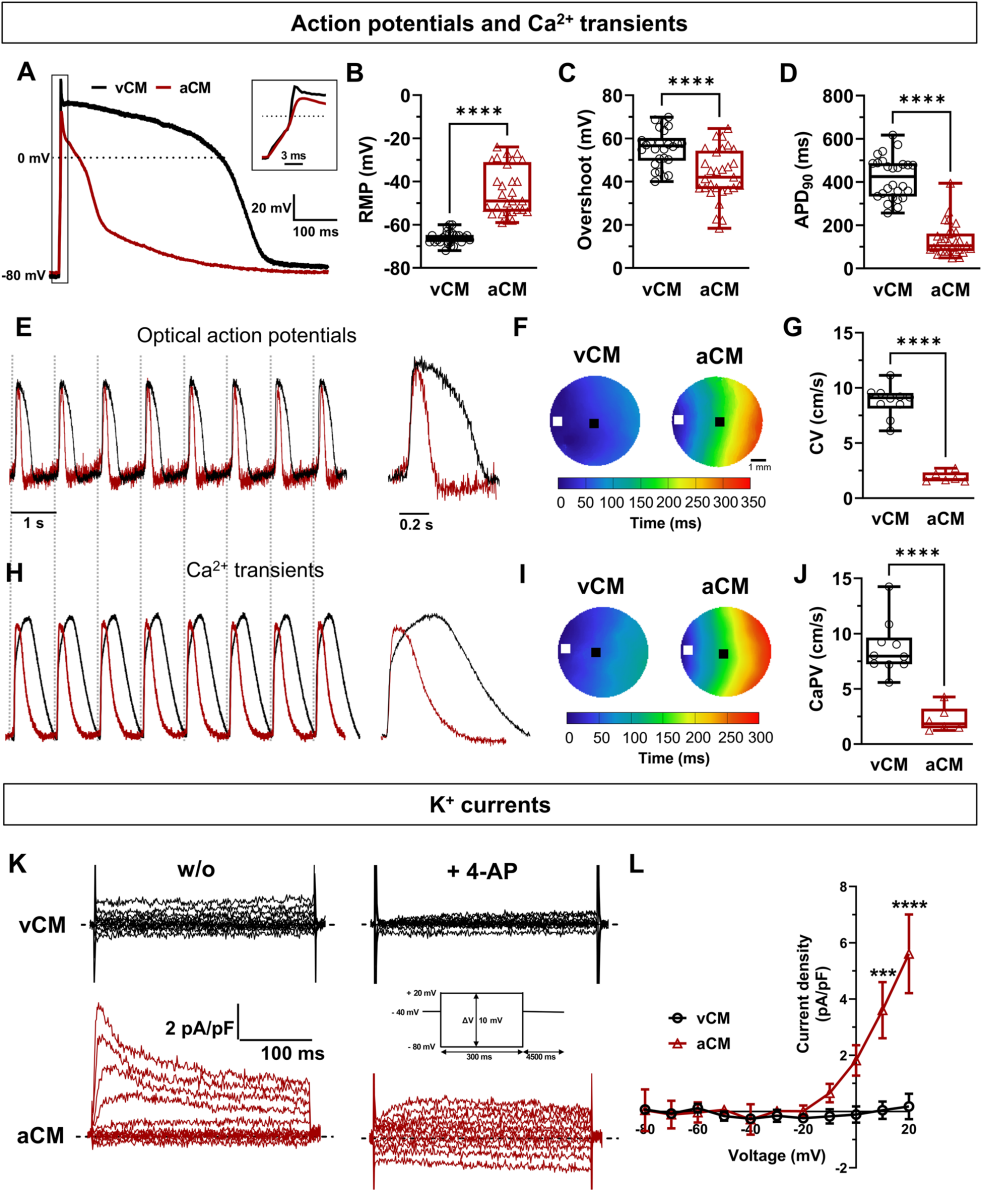
Characterization of the cardiac electrical activity, Ca^2+^ homeostasis and atrial-specific K^+^ currents in vCMs and aCMs. (**A**) Superposed APs recorded in current-clamp mode at 1 Hz. The dashed line represents 0 mV. The inset represents a magnification of the depolarizing phase of the action potential. The 3 ms scale bar represents the duration of the injected current pulse. (**B**, **C**, **D**) Box and whiskers summarizing the resting membrane potential (B), the overshoot (C), and the APD at 90% repolarization (D). (**E**, **H**) Representative optical action potentials (OAPs, E) and Ca^2+^ transients (H) simultaneously recorded in CM monolayers using RH237 and Rhod-2, respectively. The vertical dashed lines represent a stimulation at 1 Hz. (**F**, **I**) Representative activation maps at 1 Hz. The left symbol (□) indicates the position of the stimulating electrodes and the right symbol (■) indicates the position of the recordings. (**G**, **J**) Box and whiskers summarizing AP conduction (G) and Ca^2+^ propagation (J) velocities (CV and CaPV). (**K**) Representative K^+^ current densities recorded in voltage-clamp mode before and after the perfusion of 0.1 mmol/L and 1 mmol/L 4-AP, an inhibitor of voltage-gated potassium channels. The dashed line represents zero current. The inset corresponds to the patch-clamp protocol to record K^+^ currents. (**L**) Normalized I/V obtained after subtracting the K^+^ currents recorded under 4-AP perfusion from the K^+^ currents measured without 4-AP. The resulting trace corresponds to the 4-AP-sensitive K^+^ currents.

To validate atrial specification, it is essential to further study the properties of APs, atrial-specific potassium currents, and Ca^2+^ transients (CT) in vCMs and aCMs. It is already known that aCMs display shorter AP and Ca^2+^ transients than vCMs^19^. We investigated the electrical phenotypes at the single-cell and tissue levels using patch-clamp and optical mapping techniques, respectively (Fig. 2). vCMs and aCMs both triggered spontaneous APs and displayed a variable resting membrane potential (RMP) that could be identified as the maximum diastolic potential of cardiomyocytes. aCMs exhibited a more depolarized RMP (vCMs: − 66.04 ± 0.59 mV; aCMs: − 44.38 ± 2.08 mV) and a higher spontaneous beating frequency (1.42 ± 0.17 Hz) than vCMs (0.68 ± 0.06 Hz) (Fig. 2B, Suppl. Table S1) at room temperature. The following experiments were carried out on hiPSC-CMs stimulated at 1 Hz to normalize the beating rate and enable further analysis. In addition, all APs were elicited from a holding potential of -80 mV allowing us to recover some ion channels to their closed state, to generate proper APs with substantial amplitudes, and to stabilize the cells and ensure the suppression of automatic activity. As expected, vCMs and aCMs exhibited two distinct AP shapes (Fig. 2A,E). Based on a selection with the duration of the AP, 69% of vCMs displayed a ventricular-like AP shapes and 31% displayed atrial-like AP shapes. No nodal-like APs were detected. The treatment with RA shifted cell populations into 27% ventricular-like APs, 66% atrial-like APs and 9% nodal-like APs. APs and optical APs (OAPs) from aCMs exhibited a significant decrease in overshoot of 22%, upstroke velocity (dV/dT_max_) of 22%, and duration (APD) when measured at 20, 50, and 90% of repolarization compared to vCMs (Fig. 2C,D, Suppl. Table S1). Extended experiments for AP recordings were conducted using a commercially available dynamic-clamp system^22^ (Suppl. Fig. S5). This system allows the injection of a synthetic and dynamic I_K1_ current, which is lacking in hiPSC-CMs and plays a role in membrane repolarization and maintaining the RMP of cardiomyocytes. Results from this new approach confirmed the significant differences in AP parameters observed by the holding command method between aCMs and vCMs (Fig. 2). Additionally, current-clamp recordings of a second hiPSC-CM line from a male control showed that vCMs and aCMs had similar differences in AP properties, with aCMs exhibiting a decrease in overshoot, dV/dt_max_ and duration (Suppl. Fig. S6).

Activation maps for vCM and aCM monolayers from the optical recordings (Fig. 2F,G; Suppl. Table S1) notably showed that the conduction velocity (CV) was slower in aCMs (1.90 ± 0.19 cm/s) than in vCMs (8.78 ± 0.44 cm/s). The propagation of Ca^2+^ transients was measured simultaneously with OAPs using another probe on the same monolayers. As seen in the OAP recordings, the Ca^2+^ transient (CT) characteristics of aCMs monolayers were modified, including a decrease in amplitude (vCMs: 0.96 ± 0.01 A.U; aCMs: 0.91 ± 0.01 A.U), duration (CT TD80 = vCMs: 785.85 ± 15.73 ms; aCMs: 350.26 ± 30.05 ms), half time to peak (vCMs: 265.4 ± 6.0; aCMs: 51.1 ± 3.6 ms), and decay speed (τ) (vCMs: 411.8 ± 4.3 ms; aCMs: 197.1 ± 10.4 ms) (Fig. 2H, Suppl. Table S1). The activation maps show that aCM monolayers exhibited a significantly slower propagation of Ca^2+^ transients (2.26 ± 0.47 cm/s) than vCM monolayers (8.67 ± 0.77 cm/s) (Fig. 2I,J, Suppl. Table S1).

Finally, we confirmed the atrial specification of RA-treated hiPSC-CMs by investigating the atrial-specific ultrarapid delayed rectifier K^+^ current (I_Kur_), coded by the *KCNA5* gene^23^. K^+^ currents were measured in the absence and presence of a 4-AP perfusion. After subtracting the K^+^ currents in the presence of 4-AP from the potassium currents without 4-AP, we were able to determine the 4-AP-sensitive I_Kur_ currents. Our observations revealed that a low concentration of 4-AP (100 µmol/L) was effective in inhibiting I_Kur_ in aCMs, resulting in a current density at + 20 mV of 5.60 ± 1.40 pA/pF. In contrast, a high concentration of 4-AP at 1 mmol/L did not have any significant effect on currents in vCMs, indicating the absence of both I_Kur_ and the transient outward current I_to_ in these cells (Fig. 2L).

The depolarized RMP and the increase of the spontaneous beating frequency in aCMs could be explained by an implication of HCN channels. HCN channels are known to be more expressed in sinoatrial-like cardiomyocytes and atrial-like hiPSC-CMs^24^. To investigate this hypothesis, we also recorded the funny current (I_f_ current) in hiPSC-CMs (Suppl. Fig. S7). We measured a higher I_f_ current in aCMs (− 14.10 ± 1.49 pA/pF at − 125 mV) than in vCMs (− 4.88 ± 0.72 pA/pF at − 125 mV) (Suppl. Fig. S7A,B). No difference was found in the channel activation properties between aCMs and vCMs (Suppl. Fig. S7C).

Overall, these results show that hiPSC-CMs differentiated into aCMs exhibited atrial-specific markers and electrical properties, while vCMs kept their ventricular phenotype during differentiation.

### Characterization of L-type voltage-gated Ca^2+^ channels

**Figure 3:**
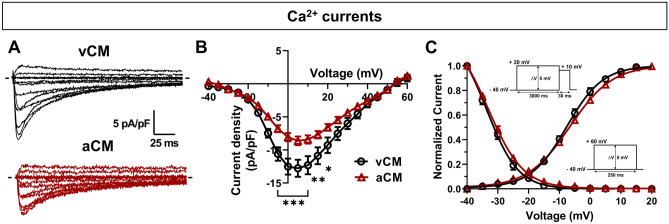
Biophysical properties of L-type voltage-gated Ca^2+^ channels in vCMs and aCMs. (**A**) Representative Ca^2+^ current densities recorded in voltage-clamp mode. The dashed line represents zero current. (**B**) Normalized intensity/voltage relationships (I/V). (**C**) Steady-state activation and inactivation of Ca^2+^ currents. The patch-clamp protocols used are inserted as insets in the graph for activation (right) and inactivation (top).

We also investigated Ca^2+^ currents by patch-clamp to evaluate differences between vCMs and aCMs (Fig. 3, Suppl. Table S2). Voltage-gated Ca^2+^ channels (VGCCs) play a crucial role in excitation–contraction coupling as they are the main route for Ca^2+^ entry into cells and are the trigger element for massive Ca^2+^ release and contraction. They thus contribute to the plateau phase, while their inactivation contributes to AP repolarization^25^.

A holding potential of − 40 mV to inactivate voltage-gated sodium channels and T-type VGCCs was imposed to record specifically L-type VGCCs. The Ca^2+^ current densities of aCMs were significantly smaller of 32% (− 8.63 ± 0.65 pA/pF) than those of vCMs (− 12.77 ± 1.29 pA/pF), with a low conductance (vCMs: 11.18 ± 1.42 pS; aCMs: 6.32 ± 0.59 pS) (Fig. 3A,B, Suppl. Table S2). No significant differences in steady-state activation and inactivation were observed. However, we detected a significant increase in the K slope factor of activation in aCMs (6.65 ± 0.27 A.U.) in comparison to vCMs (5.54 ± 0.25 A.U.) (Fig. 3C, Suppl. Table S2). In addition, the time constant of inactivation decay showed no significant difference between aCMs and vCMs (Suppl. Fig. S2E). These results demonstrated that the treatment with RA during the cardiac differentiation resulted in changes in the current density of L-type VGCCs, while having minimal effect on the gating properties of the channel.

### Biophysical properties of voltage-gated Na^+^ channels

**Figure 4:**
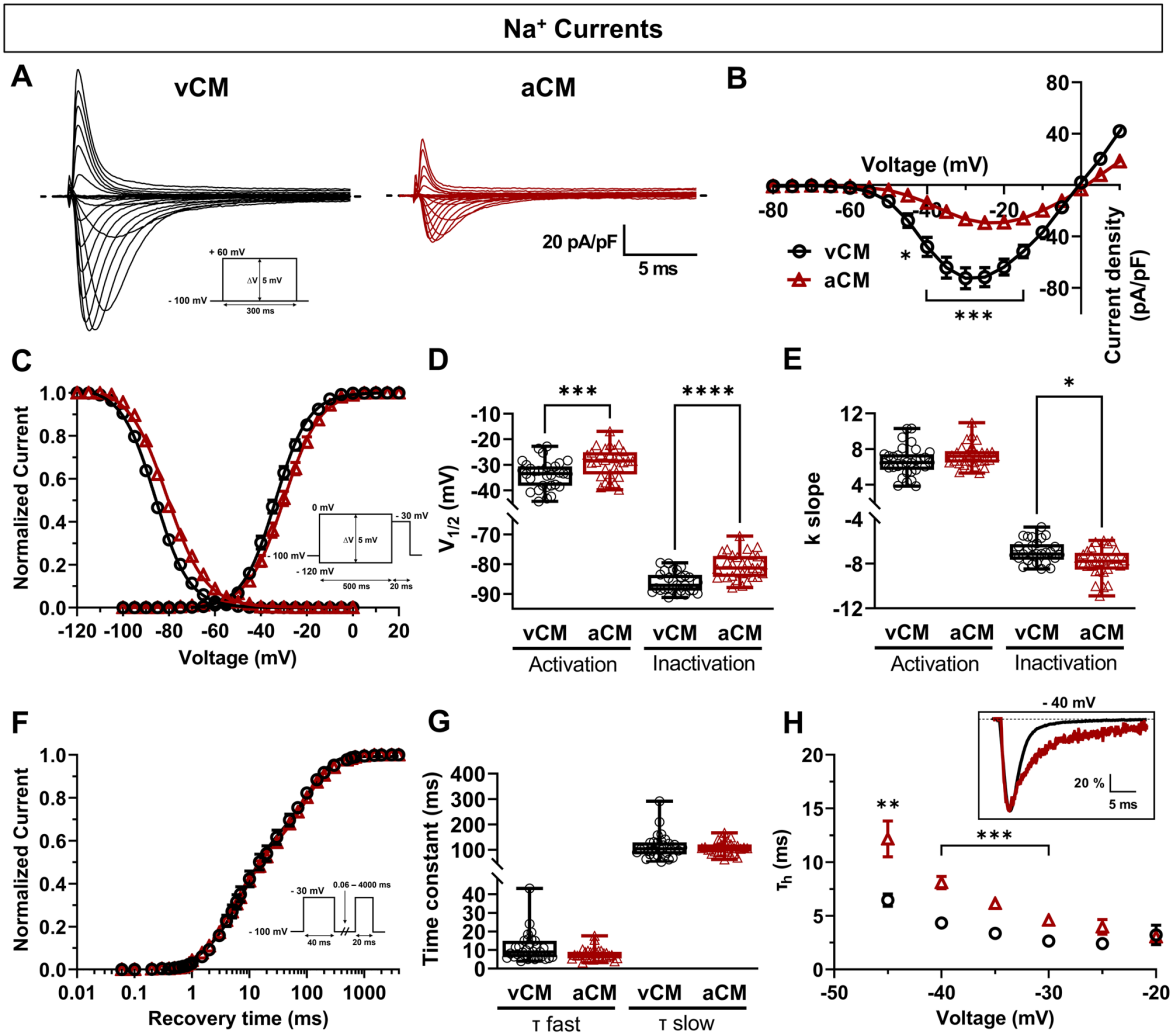
Biophysical properties of voltage-gated Na^+^ channels in vCMs and aCMs. (**A**) Representative Na^+^ current densities recorded in voltage-clamp mode. The dashed line represents zero current. (**B**) Normalized intensity/voltage relationships (I/V). (**C**) Steady-state activation and inactivation of Na^+^ currents. (**D**, **E**) Box and whiskers summarizing the half-activation and half-inactivation potentials (D) and the K slope factor (E). (**F**) Recovery from inactivation. (**G**) Box and whiskers summarizing the recovery time constants. (**H**) The time constants of fast inactivation decay plotted as a function of voltage. Inset shows the percentage of I/I_max_ as a function of time.

Na_V_1.5 plays a major role in initiating Na^+^ entry and triggering the depolarization phase of APs. Na_V_1.5 is the target of numerous drugs for the treatment of atrial- and ventricular-linked cardiac arrhythmia. This is why it is important to characterize the biophysical properties of Na_V_1.5 channels in aCMs. Interestingly, aCMs exhibited a Na^+^ current density significantly lower than that of vCMs (Fig. 4A). Indeed, the Na^+^ current density was reduced by 63% (vCMs: − 72.60 ± 8.16 pA/pF; aCMs: − 26.59 ± 3.03 pA/pF) and the conductance by 59% (vCMs: 91.51 ± 10.19 pA/pF; aCMs: 37.69 ± 4.81 pA/pF) in aCMs compared to vCMs (Fig. 4B, Suppl. Table S2). The analysis of steady-state activation and inactivation revealed significant + 4.8 mV and + 5.5 mV shifts, respectively, toward depolarized voltages in aCMs compared to vCMs (Fig. 4C,D; Suppl. Table S2). There was no significant difference in the voltage dependence of activation (K slope factor) of Na^+^ currents between vCMs and aCMs, but the voltage dependence of inactivation for aCMs was significantly lower than for vCMs (Fig. 4E, Suppl. Table S2). In addition, the window current measured was 2.35-fold higher in aCMs than in vCMs (Suppl. Fig. S3D). Measurements of the kinetics of recovery from inactivation (Fig. 4F) showed no significant differences in the fast and slow time constants of the recovery time (Fig. 4G; Suppl. Table S2). The time constant of inactivation decay showed that aCMs had a slower inactivation decay (τ_h_ at − 40 mV: 8.09 ± 0.58 ms) than vCMs (τ_h_ at − 40 mV: 4.34 ± 0.47 ms) (Fig. 4H). In parallel, we repeated these experiments with the second hiPSC-CM line differentiated with and without RA (Suppl. Fig. S8). Briefly, we observed a reduction in Na^+^ current density, an increase in V_1/2 (activation)_, and a slower inactivation decay in aCMs compared to vCMs. These data show that the atrial specification induced by RA led to a modulation of the biophysical properties of the Na_V_1.5 channel.

### Quantification of the regulatory β-subunits of the Na_V_1.5 channel

**Figure 5:**
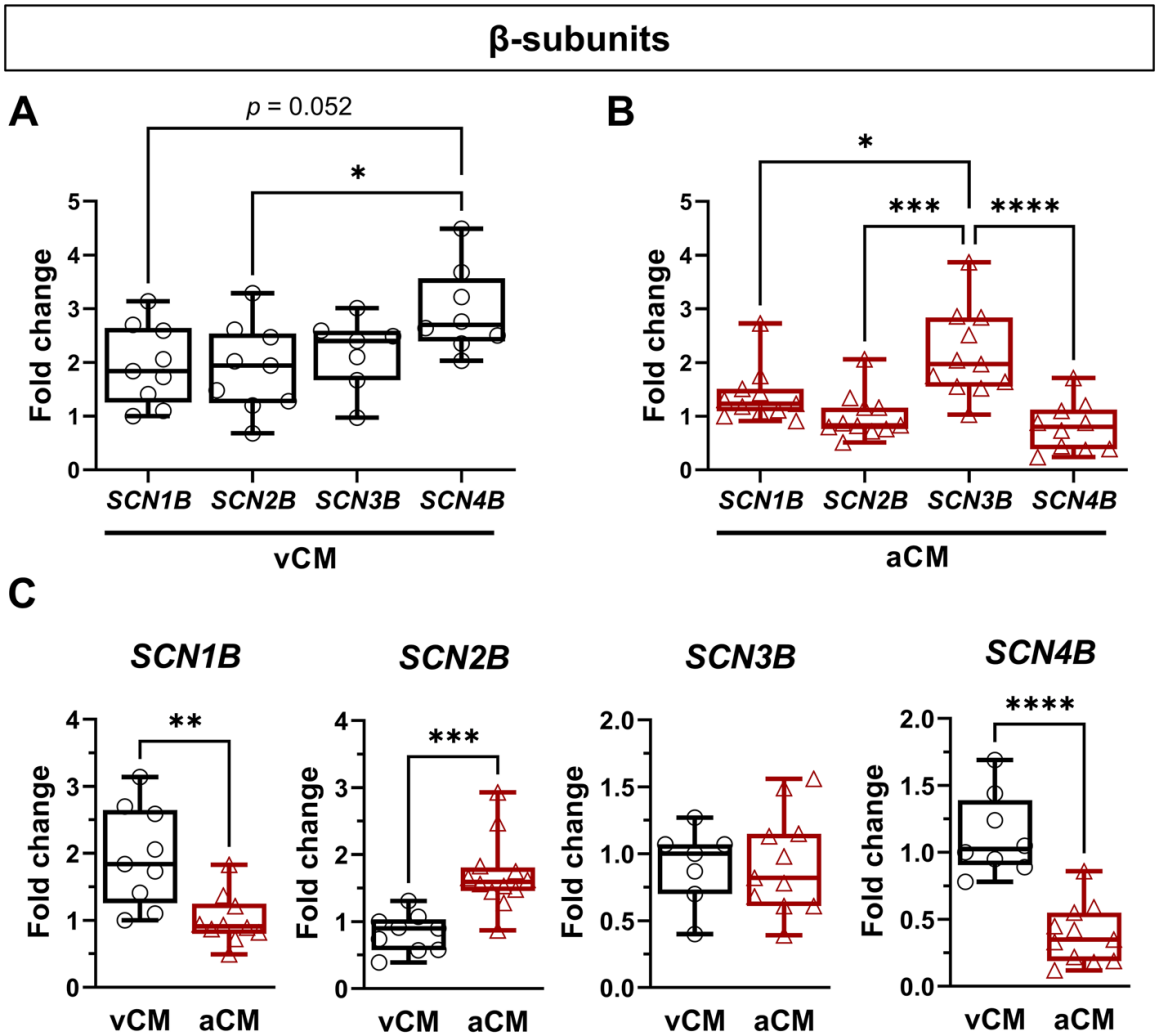
Characterization of the regulatory β-subunits of the Na_V_1.5 channel. (**A**) β-subunit expression in vCMs normalized to the expression of *SCN1B*. (**B**) β-subunit expression in aCMs normalized to the expression of *SCN1B*. All β-subunits (*SCN1B*, *SCN2B*, *SCN3B*, and *SCN4B*) of the Na_V_1.5 channel were evaluated by qPCR. (**C**) Comparison of the expression of β-subunits between vCMs and aCMs.

The differences observed in the biophysical properties of the Na_V_1.5 channel could be related to its regulatory β-subunits. Indeed, β-subunits are known to modulate Na_V_1.5 channel trafficking, cell surface expression, and gating properties^5^. We analyzed the expression of *SCN1B*, *SCN2B*, *SCN3B*, and *SCN4B* (Fig. 5). First, we normalized all β-subunit expression on housekeeping genes, and then β-subunit expression (*SCN2B*, *SCN3B* and *SCNB4)* to *SCN1B* to establish an expression profile in each condition vis-à-vis *SCN1B* (Fig. 5A,B). In vCMs, *SCN4B* was the most expressed β-subunit in comparison to *SCN1B* (1.5-fold higher) and *SCN2B* (1.6-fold higher) (Fig. 5A). In aCMs, *SCN3B* was expressed to a greater degree than *SCN1B* (1.6-fold higher), *SCN2B* (2.1-fold higher) and *SCN4B* (2.7-fold higher) (Fig. 5B). We then compared β-subunit expression between vCMs and aCMs, and we observed a higher expression of *SCN2B* (2.1-fold higher) and a lower expression of *SCN1B* (1.9-fold lower) and *SCN4B* (2.9-fold lower) in aCMs (Fig. 5C). No significant difference was found for *SCN3B* (Fig. 5C). These data show that β-subunits were differentially expressed in vCMs and aCMs, potentially contributing to the differences observed in the biophysical properties of Na_V_1.5 channels.

## Discussion

In the present study, we investigated the effect of the atrial specification of hiPSC-CMs produced by a RA treatment on the biophysical properties of Na_V_1.5 channels. The RA treatment enabled us to obtain hiPSC-CMs expressing atrial-specific markers with an electrophysiological profile close to mature atrial CMs. In addition, aCMs exhibited differences in their ionic current properties. An extended analysis of Na_V_ channels revealed that the Na^+^ current density was reduced, with changes in the biophysical properties that can be associated with modifications in β-subunit expression.

In our cell model, we induced atrial specification with a RA treatment. Indeed, RA has been well-known to drive the atrial specification of hiPSC-CMs^19–21,26^. In this context, we decided to apply RA directly with the commercial cardiac differentiation kit from StemCell company, because it is a reliable and well-control way to obtain homogenous and reproductible cardiac differentiation. We based our protocol on the previous well established and published protocols, and we added the RA directly into the commercial protocol to keep its advantages. We applied this approach to ensure that our aCMs had an atrial phenotype. The aCMs exhibited an upregulation of atrial and sinoatrial markers such as *MYL7* and *CACNA1D*, and a downregulation of ventricular markers such as *GJA1* and *MYL2*. These results were in accordance with other studies using RA as modulator of atrial differentiation^19,20^. Furthermore, these shifts in gene expression, particularly in the *MYL7/MYL2* genes, were found to align with trends observed in human atrial and ventricular tissues (Data not shown).

Moreover, AP recordings of single cells and monolayers allowed us to evaluate the electrophysiological profiles of hiPSC-CMs. Single-cell AP recordings were conducted using two different methods to set a holding potential close to − 80 mV for a rigorous comparison of AP parameters. The holding command method involves the injection of a constant current to polarize the membrane potential of cells to a more negative value. However, this approach can affect the APD of cardiomyocytes^27^. Dynamic clamp is a technique that electronically introduces ionic currents, such as I_K1_, into cells to compensate for the absence of their natural expression, resulting in more stable AP recordings in hiPSC-CMs. Both methods confirmed that the APD of aCMs was shorter than that of vCMs with a more triangular AP shape. A study in human heart demonstrated that the APD_90_ values are comparable between atrial and ventricular tissues^28^. However, in the same study, they also observed a decrease in APD_50_ in atrial myocytes compared to ventricular myocytes. These atrial myocyte APs exhibited a more triangular shape. In the context of hiPSC-CMs, numerous studies have consistently shown that the APD_90_ is drastically shorter in atrial hiPSC-CMs compared to ventricular hiPSC-CMs, with a triangular-like shape^16,19,20,24^. Nonetheless, it is worth noting that another study has reported a small decrease in APD_90_ between atrial and ventricular hiPSC-CMs^21^. Interestingly, the shape of these APs resembles those obtained from human myocytes, characterized by a pronounced triangular shape^28^. These differences between hiPSC-CMs and human myocytes are potentially due to the lack of maturity of hiPSC-CMs. In this work, we used AP durations to classify hiPSC-CM populations. The analysis of atrial-like and ventricular-like APs based on APD_90_ confirmed the shift of ventricular-like cardiomyocyte population towards atrial-like cardiomyocytes with the RA treatment. The treatment with RA induced the apparition of a small proportion of nodal-like cardiomyocytes (9%) but atrial-like cardiomyocytes remain in the majority (66%). Therefore, the RA treatment did not generate pure atrial cells but rather shifted the proportion of atrial and ventricular-like cells, with the majority of cells exhibiting an atrial-like phenotype. This observation is consistent with previous studies that have shown the effectiveness of RA in promoting the differentiation into atrial-like CMs^19–21,24^. Moreover, the presence of ultrarapid delayed rectifier K^+^ current (I_Kur_), a characteristic of atrial cardiomyocytes^23,29^, was confirmed in our aCMs through perfusion of the K^+^ channel inhibitor 4-AP. Indeed, the low concentration used selectively affected I_Kur_ without affecting I_to_^30,31^. In contrast, high-concentration 4-AP treatment on vCMs revealed the absence of both I_Kur_ and I_to_, consistent with previous findings^32^. However, no difference in KCNA5 protein expression was observed between aCMs and vCMs. KCNA5 protein may not be abundantly expressed in hiPSC-CMs as it was challenging to detect in comparison to other proteins, raising the possibility that the detection level was insufficient for a clear comparison of KCNA5 protein expression between aCMs and vCMs.

In addition, GJA1 proteins were localized in the perinuclear area in vCMs and aCMs whereas gap junctions of GJA1 were only identified at the plasma membrane of vCMs. In mature CMs, GJA1 proteins are mainly expressed in ventricles^33^, and a higher expression of GJA1 in CMs leads to a higher AP conduction velocity and Ca^2+^ transient propagation^34^. The perinuclear localization of GJA1 proteins participates to its lifecycle and could represent the synthesis site of the GJA1 protein in the reticulum endoplasmic/Golgi apparatus^35^. In the present study, the AP and Ca^2+^ transient propagations were faster in vCMs. This could have resulted in the higher expression of GJA1. There was also a 22% decrease of the overshoot and the dV/dt_max_ of APs in aCMs. These decreases are probably correlated to the decrease in Na_V_1.5 channel expression and its currents of more than 50%. Therefore, the smaller Na^+^ current observed in aCMs would intuitively lead to a larger decrease in the overshoot and the upstroke velocity, but the compensatory effect of the positive shift in inactivation voltage can help mitigate this decrease to some extent. By shifting the inactivation curve towards more depolarized voltages, a larger proportion of Na^+^ channels in aCMs remain available for activation during the upstroke phase of the AP. Furthermore, the increase of window current in aCMs, suggests a higher channel opening probability in aCMs than vCMs (Suppl. Fig. S3D). This availability of Na^+^ channels can help maintain a sufficient Na^+^ influx during the depolarization phase, which contributes to the preservation of the overshoot and the dV/dt_max_ to some degree. aCMs also exhibited a more depolarized RMP than vCMs. In the literature, studies on hiPSC-CMs have reported that there is no difference in RMP between vCMs and aCMs^19,23^. Nevertheless, the RMP of mature mammalian CMs is more depolarized in atrial cardiomyocytes than in ventricular cardiomyocytes^4^. In addition, the depolarized RMP may be attributed to the reduced expression of Kir channels responsible for I_K1_ currents^36^, but also to the presence of HCN channels responsible for I_f_ currents which are known to be present in hiPSC-CMs^37^. A study has shown that sinoatrial-like and atrial-like hiPSC-CMs expressed higher *HCN4* than ventricular-like hiPSC-CMs, and were sensitive to ivabradine, a specific inhibitor of I_f_ current^24^. In human heart, HCN4 proteins are strongly expressed in the sinoatrial node (SAN), atrioventricular node (AVN) and His-Purkinje system, but none could be identified in the surrounding atrial tissue^38^. A significant increase in I_f_ currents has been observed in aCMs when compared to vCMs, which likely contribute to the depolarized state observed in aCMs^37^. This finding is possibly resulted from RA treatment, and may not be connected with a decreased differentiation state in aCM. Indeed, an analysis of cardiac marker TNNT2 showed that *TNNT2* mRNA transcripts were expressed at same level in both cell types and TNNT2 protein level is higher in aCMs than in vCMs. Several studies have shown that RA treatments do not affect the cardiac differentiation of hiPSC-CMs^19,20^. However, it has also been shown that a RA treatment can favor cardiomyocyte differentiation, with an increase in the expression of TNNT2 and NKX2.5, two transcription factors implicated in the transcription of numerous important cardiac differentiation genes^24,39^. We also observed an increase in RYR2 protein expression in aCMs. RYR2 is a calcium release channel critical for excitation–contraction coupling in cardiomyocytes. The higher expression of RYR2 in aCMs suggests a more developed calcium handling machinery, which could also serve as an indicator of maturation.

An analysis of Ca^2+^ transients and L-type Ca^2+^ currents showed that Ca^2+^ influx in aCMs were reduced. The significant decrease of the *CACNA1C* expression observed by qPCR in aCMs could explained the reduction in Ca^2+^ currents and Ca^2+^ transient amplitudes observed in these cells. Nevertheless, no difference in CACNA1C protein expression was detected between the two cell types. This discrepancy may arise from potential issues with the antibody's specificity for CACNA1C or the involvement of different regulatory mechanisms governing calcium channel opening and gating properties. Nevertheless, electrophysiological recordings of calcium currents on hiPSC-CMs allowed to record functional part of L-type Ca^2+^ currents located at the plasma membrane, and they elicited a decrease of Ca^2+^ currents in aCMs. In the literature, studies have shown a reduction of *CACNA1C* gene expression and a decrease of Ca^2+^ current density associated to a shift of activation toward more depolarized voltages in both atrial adult and hiPSC-CMs^19,40^. Our observations are in accordance with these ones. In our model of atrial cardiomyocytes, we also found that *CACNA1D* gene expression increased in aCMs. In normal human adult heart, it is known that CACNA1D is not expressed in ventricles, but predominantly expressed in supraventricular tissues, including the atria, as well as in the sinus node^41^. In this regard, the role of CACNA1D in atrial cardiomyocytes was determined in a Ca_V_1.3 KO mouse model^42^. Indeed, the absence of Ca_V_1.3 channels led to the generation of atrial arrhythmia with no effect on ventricles and these results validated the importance of CACNA1D in the functionality of atrial cardiomyocytes. Furthermore, Ca_V_1.3 channels have been reported to be implicated in cardiac automaticity and rhythmicity^43^ while a Ca_V_1.3 KO decreases the cardiac beating frequency. In addition, we also observed an increase of the nodal-like cardiomyocyte population in aCMs which may also account for the elevated expression of CACNA1D. Based on these outcomes, the higher expression of *CACNA1D* in our aCMs could participate in the increase in the spontaneous beating frequency and are additional proofs of the atrial specification of our aCMs. Moreover, it has been shown that the beating frequency was reduced by the application of ivabradine, which confirm the importance of the I_f_ current in the rhythmicity^24^. Therefore, the increase of the spontaneous beating frequency in aCMs could be also explained by the implication of HCN channels.

Thus far there have been no functional explorations of the electrophysiological profile of Na_V_ channels (or Na^+^ currents) in hiPSC-CMs differentiated into aCMs with a RA treatment. Nevertheless, several studies have compared the biophysical properties of Na_V_1.5 channels in an adult human and animal model of CMs^2,3,6,7,44–46^. No significant difference was found in *SCN5A* transcript expression in mature human atrial and ventricular cardiomyocytes^33,47^ but this information was not validated on mouse heart cardiomyocytes where authors showed an increase in Na_V_1.5 channel protein expression in the left atria in comparison to the left ventricle^4^. Furthermore, several studies have shown that Na^+^ current densities are higher in mature atrial cardiomyocytes than in mature ventricular cardiomyocytes. These observations were conducted on canine myocytes^44,46^, rabbit myocytes^2^, rat myocytes^3,45^ and human myocytes^6, 7^. Sakakibara and his colleagues noticed a 2.5-fold increase of the Na^+^ current density in human atrial cardiomyocytes than in human ventricular cardiomyocytes. However, it is worth noting that no significant difference was found in Na conductance between human atrial and ventricular cardiomyocytes in this investigation^48^. These findings offer evidence for chamber-specific differences in I_Na_ in the human heart. Nevertheless, these results are not in agreement with the decrease in Na_V_1.5 expression and current density in aCMs observed in the present study. In addition, the Na_V_1.5 expression and its channel activity are strongly implicated in AP propagation within cardiomyocytes^9^. Therefore, the strong reduction of Na_V_1.5 channel expression and Na^+^ current density could contribute to the decrease of the conduction velocity observed in aCMs. These differences in Na^+^ current density could be explained by variations in β-subunit expression. Indeed, SCN1B and SCN3B subunits are known to be implicated in Na_V_1.5 channel expression and can modulate the current density^5,49,50^. In our CMs, *SCN1B* exhibited lower expression in aCMs compared to vCMs. These findings do not align with previous studies that demonstrated, in SCN1B knock-out mouse models, that the deletion of SCN1B protein led to an increase in Na^+^ current densities without affecting gating properties^49,50^. Consequently, the observed decrease in Na^+^ current densities and alterations in sodium channel gating properties in aCMs were likely unrelated to the expression of the *SCN1B* gene. Furthermore, Zhu and her colleagues^49^ revealed that *SCN1B* transcripts were more highly expressed in human atrial cardiomyocytes than in human ventricular cardiomyocytes. These results contrast with the *SCN1B* expression levels found in hiPSC-CMs. In addition, it appears that β-subunits are not expressed at the same level as in mature human or mammalian cardiomyocyte models. It is worth noting that the expression pattern of β-subunits in hiPSC-CMs resembles that of embryonic hearts rather than adult hearts^51^. These observations highlight the distinct expression profile of β-subunits between hiPSC-CMs and adult cardiomyocytes. Given these results, it is not surprising to observe disparities in the expression of β-subunits between our hiPSC-CM model and adult cardiomyocytes. These disparities between human cardiomyocytes and hiPSC-CMs could be explained by the immaturity state present in hiPSC-CMs. However, they could also be influenced by variations in the proportion of cell subtypes within hiPSC-cardiomyocyte populations. We also detected a low expression of GJA1 proteins in aCMs and it was reported that a strong relationship exists between the expression levels of the GJA1 and Na_V_1.5 proteins^52^. Indeed, a loss of GJA1 expression leads to a reduction of Na_V_1.5 expression associated with a decrease in Na^+^ current density. Moreover, Sottas et al. have reported that the overexpression of GJA1 in hiPSC-CMs led to an increase in Na^+^ current density^34^. Numerous other processes may be involved in the modulation of the biophysical properties of Na_V_1.5 channels, including glycosylation, phosphorylation, and partner proteins^5^. When we characterized the biophysical properties of Na_V_1.5 channels, we also observed a shift in activation and inactivation toward more depolarized voltages. The SCN2B and SCN4B subunits are also implicated in the modulation of the gating properties of Na_V_1.5 channels^5^. SCN2B and SCN4B are more highly expressed in rat vCMs than in aCMs^45,49^. These authors also reported that there is a shift in activation and inactivation toward more depolarized potentials. The overexpression of hNa_V_1.5 with hSCN2B/hSCN4B subunits in a heterologous system confirms these results. In our model, activation and inactivation were shifted toward more depolarized potentials in aCMs. In addition, aCMs expressed more *SCN2B* and less *SCN4B* subunits. Therefore, these results lead us to believe that the *SCN2B* subunit could be mainly responsible for the differences in Na_V_1.5 gating properties observed in vCMs and aCMs. Furthermore, approximately 98–99% of Na_V_1.5 isoforms were composed of neonatal isoforms in our hiPSC-CMs (Suppl. Fig. S3B), and only 1–2% could be composed of the adult isorforms. A study of Onkal and his collaborators showed that the adult and the neonatal isoforms of Na_V_1.5 presented different biophysical properties including changes in their activation and inactivation^53^. Knowing that the neonatal isoform is largely expressed in both cell population, differences observed in the biophysical properties of Na_V_1.5 between vCMs and aCMs cannot be explained by the proportion of adult and neonatal isoforms. Nevertheless, there are two times more adult *SCN5A* isoforms in aCMs than in vCMs (Suppl. Fig. S3C), and this information supports the idea that aCM differentiation seems to be unimpaired, and aCMs are not lacking maturity in comparison to vCMs. Numerous studies have also described a shift in the activation and inactivation of Na_V_1.5 toward more polarized potentials in mature aCMs^2,3,44,45^. These differences in the biophysical properties of Na_V_1.5 could explain the differential sensitivity to certain class I antiarrhythmic drugs (ranolazine, flecainide, lidocaine) used to treat atrial arrhythmogenic disorders^2,4,44^. In this study, we aimed to explore the biophysical properties of the Na_V_1.5 channel in hiPSC-CMs. This cell model has gained widespread usage in the field of cardiac research, as it offers several advantages such as human origin, accessibility, easy to cultivate, avoidance of ethical concerns, and the ability to differentiate into various cell types. Our investigation revealed that the electrophysiological characteristics of aCMs and vCMs did not precisely mirror those observed in mature cardiomyocyte models. This highlights the necessity of performing electrophysiological characterization, specifically on hiPSC-based models. Additionally, these results have implications for studies seeking to better understand channelopathies or develop therapeutic interventions. In the future, it could also be interesting to investigate K^+^ channels, including I_Kr_, I_Ks_ and I_K1_, as well as more specific atrial K^+^ channels such as I_Kach_ and I_SKCa_ in order to gain insights into their expression and functional properties in aCMs.

### Study limitations

This study uncovers novel insights into the activity of ion channels in hiPSC-CMs both when differentiated with and without RA. RA has proven effective in generating hiPSC-CMs with an atrial-like phenotype. However, numerous disparities emerge when comparing these results to mature models of cardiomyocytes derived from animals or humans. These differences likely stem from the immature phenotype characteristic of hiPSC-CMs. Moreover, hiPSC-CMs consist of a heterogeneous mixture of cells with varying phenotypes and differentiation stages, complicating the analysis when compared to mature models. Additionally, RA is also known to increase the population of sinoatrial-like cardiomyocytes in hiPSC-CMs. Hence, it is crucial to take into account that the existence of aCMs may impact certain observations. Investigating the potential synergy of RA with other factors to improve atrial differentiation is a subject that deserves further explorations in the future.

## Conclusion

The results reported here highlight the interest of using RA to generate functional atrial-like hiPSC-CMs. This approach enabled us to characterize various ion channels particularly voltage-gated Na^+^ channels in aCMs, which exhibited expression and specific biophysical properties that were different from those of ventricular-like cardiomyocytes. The analysis of regulatory β-subunit expression of the Na_V_1.5 channel allowed us to understand the differences observed in the biophysical properties of the channel. These hiPSC-CMs represent an abundant source and continue to hold promise for the development of ventricular-specific and atrial-specific ion channel modulators, as well as for modeling ventricular-specific and atrial-specific diseases.

## Material and methods

### hiPSC cultures and cardiomyocyte differentiation

The hiPSC lines CBRCULi001-A^54^ and CBRCULi008-A^55^ were generated from a 44-year-old male and 75-year-old female control lymphoblastoids, respectively, and they were reprogramed at the LOEX core facility (Quebec City, QC, Canada). All the work with hiPSCs were approved by CIUSSS de la Capitale-Nationale ethics committee (Project #2019-1734). All methods were carried out in accordance with relevant guidelines and regulations and were conducted by respecting approved protocols by the ethics committee. Furthermore, these procedures were only performed after obtaining informed consent from both patients. The hiPSC line CBRCULi008-A was used for all experiments and the hiPSC line CBRCULi001-A for experiments indicated in the supplementary data. They were grown on hESC-qualified Matrigel (Cat# 354277, Corning, AZ, USA) in mTeSR plus medium (Cat# 100-0276, STEMCELL Technologies, BC, Canada) and were routinely dissociated using 500 μM EDTA every 4–6 days. They were then differentiated into ventricular (vCMs) and atrial cardiomyocytes (aCMs). The hiPSCs were differentiated into ventricular cardiomyocytes (vCMs) with a monolayer-based protocol using STEMdiff™ Ventricular Cardiomyocyte Differentiation kits (Cat# 05010, STEMCELL Technologies) according to the manufacturer’s protocols and instructions. Spontaneously beating cells were observed at day 8 to 12 of differentiation. The hiPSCs were also differentiated into atrial cardiomyocytes (aCMs) by adding 1 μmol/L all-trans retinoic acid (RA) between 2 and 5 days of differentiation (Cat# 72262, STEMCELL Technologies). The hiPSC-CMs were maintained in STEMdiff™ Cardiomyocyte Maintenance medium (Cat# 05020, STEMCELL Technologies) until they reached 30 days of maturation. Only the efficient differentiations with a spontaneously beating monolayers, were selected to perform the experiments.

### Gene expression analysis

RNA was extracted from the hiPSC-CMs on day 30 of differentiation using Quick-RNA MiniPrep kits (Cat# R1054, Cedarlane, ON, Canada), and cDNA was synthesized by the QuantiTect Rev. Transcription Kit protocol (Cat# 205313, Qiagen, Hilden, Germany). qPCR assays were performed using SYBR green I detection dye on an LC480 platform (Roche, Basel, Switzerland) using the seller’s specifications. The primers are listed in Supplementary Table S3. All the qPCR reactions were run in triplicate with a non-template control (NTC). qPCR efficiencies were obtained using a series of cDNA dilutions and were calculated using the slope of the regression line determined using the following equation: E = 10 [− 1/slope]. All qPCR reactions had an efficiency ranging from 1.7 to 2.3. The analyses were performed using LightCycler^®^ 480 SW 1.5 software. Run-to-run variations were adjusted using a known standard, and quantifications were corrected for efficiency. The specificity of the amplification for each run was controlled using a melting curve analysis. The quality of the differentiation was assessed by measuring the *TNNT2* gene level. All the samples with the lowest cycle threshold (CT) values were totally removed. When comparing vCM and aCM conditions for a specific gene, normalization was carried out by dividing the CT of aCM by that of vCM. To facilitate comparisons across different genes, normalization was performed with respect to a single gene. Gene expression's relative quantification was executed using the ΔΔCT method. For standardization purposes, the obtained relative abundance value was divided by the mean value obtained from two housekeeping genes (*RPL22*, *PPIA*). For the *SCN5A* mRNA analysis, targeting the exon 25 of *SCN5A* mRNA covered all isoforms, including the adult (exon 6b) and “neonatal” (or fetal, exon 6a) sodium channel isoforms. The percentage of adult isoform was obtained using the following ratio: isoform adult (exon 6b)/isoform neonatal (exon 6a) × 100.

### Western blotting

The proteins of the hiPSC-CMs were extracted on day 30 of differentiation by scraping the cells into Radioimmunoprecipitation Assay buffer (RIPA buffer: 50 mmol/L Tris–Cl, 1 mmol/L EDTA, 150 mmol/L NaCl, 0.5% SDS, 1% NP-40) supplemented with proteases (Cat# 5892970001, Sigma-Aldrich) and phosphatase inhibitor cocktails (Cat#4906845001, Sigma-Aldrich). The lysate was incubated for 2 h at 4 °C under gentle rotation and was clarified by centrifugation at 18,000 g for 5 min at 4 °C. Protein concentrations were measured using Pierce™ BCA Protein Assay kits (Cat# 23225, ThermoFisher Scientific) with a bovine serum albumin (BSA) standard range (20 to 2000 µg/mL) as a reference. Protein extracts (20 μg) were denatured in 5X sample buffer (156 mM Tris–Cl, pH 6.8, 0.025% bromophenol blue, 5% SDS, 50% glycerol, 12.5% β-mercaptoethanol) at 37 °C for 30 min. They were resolved on 4–15% Mini-PROTEAN^®^ TGX Stain-Free™ Protein gels (Cat# 456-8083, Bio-Rad) and were blotted on 0.45-μm PVDF membranes with Trans-Blot Turbo RTA Midi 0.45 µm LF PVDF Transfer kits (Cat# 1704275, BioRad). The PVDF membranes were blocked and were incubated with rabbit anti-sodium voltage-gated channel alpha subunit 5 (SCN5A) (1:200, Cat# ASC-005, Alomone Labs, RRID:AB_2040001), rabbit anti-calcium voltage-gated channel subunit alpha 1 C (CACNA1C) (1:200, Cat# ACC-003, Alomone Labs, RRID:AB_2039771), mouse anti-myosin light chain 7 (MYL7) (1:400, Cat# ab68086, Abcam, RRID:AB_1140497), rabbit anti-myosin light chain 2 (MYL2) (1:2000, Cat# ab79935, Abcam, RRID:AB_1952220), mouse anti-TNNT2 (1:5000, Cat# ab10214, Abcam, RRID:AB_2206574), rabbit anti-gap junction protein alpha 1 (GJA1) (1:5000, Cat# ab11370, Abcam, RRID: AB_297976), rabbit anti-potassium voltage-gated channel subfamily A member 5 (KCNA5) (1:200, Cat# APC-150, Alomone Labs, RRID: AB_10918640), rabbit anti-ryanodine receptor 2 (RYR2) (1:1000, Cat# ARR-002, Alomone Labs, RRID: AB_2040184), or rabbit anti-glyceraldehyde 3-phosphate dehydrogenase (GAPDH) (1:20,000, Cat# A300-641A, Bethyl, RRID:AB_513619). Goat horseradish peroxidase (HRP)-conjugated anti-rabbit (1:10,000, Cat# 111-035-003, Jackson ImmunoResearch, RRID:AB_2313567) and anti-mouse (1:10,000, Cat# 115-035-003, Jackson ImmunoResearch, RRID:AB_10015289) were used as secondary antibodies. Proteins were revealed using Clarity and Clarity Max Western ECL substrates(Cat# 1705060 and Cat# 1705062, Bio-Rad) and were visualized using the ChemiDoc system (Bio-Rad, ON, Canada). Relative quantification of protein expression was performed with ImageJ software. The total intensities of the bands of the targeted proteins were divided by the normalization factor obtained after dividing the observed signals of total protein in the lane with the strongest observed signal of total protein on the blot. Images of all the gels and blots are available in Suppl. Fig. S4.

### Immunocytofluorescence staining

hiPSC-CMs were dissociated at D23 using STEMdiff™ Cardiomyocyte Dissociation Medium (Cat# 05025, STEMCELL Technologies) and were seeded on Matrigel-coated 12-mm glass coverslips in a 24-well plate at a density of 50,000 cells/cm^2^. At D30, the cells were fixed with 4% paraformaldehyde, permeabilized, and saturated in a PBS solution containing 0.2% Triton X-100, 5% goat serum, and 1% BSA for 30 min. hiPSC-CMs were stained overnight at 4 °C using a blocking solution (1% BSA / 5% GS in PBS) containing the following primary antibodies: human anti-TNNT2-FITC (1:200, Cat# 130-119-674, Miltenyi Biotec, RRID:AB_2751795), mouse anti-actinin alpha 1 (ACTN1) (1:500, Cat# ab9465, Abcam, RRID:AB_307264), rabbit anti-GJA1 (1:500, Cat# ab11370, Abcam, RRID:AB_297976), rabbit anti-MYL2 (1:100, Cat# ab79935, Abcam, RRID:AB_1952220), and human anti-MYL7-PE (1:20, Cat# 130-117-546, Miltenyi Biotec, RRID:AB_2751399). The secondary antibodies (goat anti-mouse Cy3 (1:500, Cat# A10521, Invitrogen, RRID:AB_2534030) and goat anti-rabbit AlexaFluor™ 633 (1:500, Cat# A21071, Invitrogen, RRID:AB_2535732) were then added to the blocking solution. The mixtures were incubated for 2 h at room temperature in the dark. After washing, the glass coverslips were slide mounted in Fluoromount-G mounting medium with 4′,6-diamidino-2-phenylindole (DAPI) (Cat# 00-4959-52, Invitrogen). The immunolabeled samples were acquired at × 20 objective using a Zeiss LSM780 confocal laser scanning microscope (Zeiss, Germany) and examined with ZEN software (Zeiss, Germany) and adapted with ImageJ software (NIH, Bethesda, MD, USA).

### Electrophysiology

Patch-clamp experiments were performed at room temperature using an Axopatch 200B amplifier and pClamp software v10 (Molecular Devices, CA, USA). Macroscopic Na^+^, Ca^2+^ and K^+^ currents and APs were recorded using the whole-cell configuration of the patch-clamp technique in voltage-clamp and current-clamp modes, respectively. The pipettes were drawn from borosilicate glass capillaries (Sutter Instrument, CA, USA) and were fire polished.

For the voltage-clamp experiments, the pipettes were coated with HIPEC (Dow-Corning, MI, USA) to minimize electrode capacitance. For Na^+^ currents, the pipettes were filled with a solution containing (in mmol/L) 35 NaCl, 105 CsF, 10 EGTA, and 10 HEPES. The pH was adjusted to 7.4 with CsOH. The bath solution contained (in mmol/L) 105 NMDG, 35 NaCl, 2 KCl, 1.5 CaCl_2_, 1 MgCl_2_, 10 D-glucose, 10 HEPES, 10 TEA-Cl, and 0.01 nifedipine^56^. The pH was adjusted to 7.4 with methanethiosulfonic (MTS) acid. For Ca^2+^ currents, the pipettes were filled with a solution containing (in mmol/L) 25 NaCl, 105 CsCl, 1 MgCl_2_, 10 EGTA, and 10 HEPES. The pH was adjusted to 7.2 with CsOH. The bath solution contained (in mmol/L) 100 NaCl, 5 CsCl, 5 CaCl_2_, 40 NMDG, 1 MgCl_2_, 10 D-glucose, 10 HEPES, and 15 TEA-Cl. The pH was adjusted to 7.4 with methanesulfonic acid (MSA). For K^+^ currents, the pipettes were filled with a solution containing (in mmol/L) 5 NaCl, 5.4 KCl, 136 KMeSO_4_, 5 EGTA, 5 MgATP, 5 Phosphocreatine, 1 MgCl_2_, and 1 HEPES. The pH was adjusted to 7.2 with KOH. The bath solution contained (in mmol/L) 136 NaCl, 5.4 KCl, 1.8 CaCl_2_, 1 MgCl_2_, 0.33 NaH_2_PO_4_, 5 HEPES, and 10 D-glucose. The pH was adjusted to 7.4 with NaOH. 20 µmol/L Tetrodotoxin (Cat# L8503, Latoxan, France), and 10 µmol/L Nifedipine (Cat# N7634, Millipore Sigma) were added to inhibit voltage-gated sodium channels and voltage-gated calcium channels, respectively. To study the effect of 4-aminopyridine (4-AP, Cat# 104570050, ThermoFisher Scientific) on K^+^ currents, 4-AP was added in the bath solution and perfused directly on the hiPSC-CM with a 4-channel perfusion system (Automate Scientific). A low concentration of 4-AP was used on aCMs (0.1 mmol/L) and a higher dose on vCMs (1 mmol/L). 4-AP-sensitive currents were obtained after the subtraction of K^+^ currents recorded under 4-AP treatment to K^+^ currents recorded without 4-AP treatment. Series resistance and cell capacitance were corrected. Currents were filtered at 5 kHz, digitized at 10 kHz, and stored on a microcomputer equipped with an AD converter (Digidata 1440A, Molecular Devices). P/4 leak subtraction was used prior to applying pulse stimulations for Na^+^ and Ca^2+^ current recordings.

For I_f_ currents, the pipettes were filled with a solution containing (in mmol/L) 130 K-aspartate, 10 NaCl, 10 HEPES, 5 EGTA, 2 CaCl_2_, 2 MgCl_2_, 0.1 Na-GTP, 5 Na_2_-GTP, 5 Phosphocreatine. The pH was adjusted to 7.2 with KOH. The bath solution contained (in mmol/L) 140 NaCl, 5.4 KCl, 5 HEPES, 1.8 CaCl_2_, 1 MgCl_2_, 1 BaCl_2_, 2 MnCl_2_, 5.5 D-Glucose. The pH was adjusted to 7.4 with NaOH.

Na^+^ currents were obtained using 200-ms pulses from − 100 to + 60 mV in + 5 mV increments. Ca^2+^ currents were obtained using 250-ms pulses from − 40 to + 60 mV in + 5 mV increments. K^+^ currents were obtained using 300-ms pulses from − 80 to + 20 mV in + 10 mV increments. I_f_ currents were obtained using 10-s pulses from − 35 to -125 mV in − 10 mV increments, followed by a 1-s pulse at − 125 mV. The densities were measured by normalizing current amplitudes to membrane capacitance. Activation curve for I_f_ currents was determined from the tail currents. Activation curves for Na^+^, Ca^2+^ and K^+^ currents were generated using the following standard Boltzmann distribution:

$$\frac{G\left( V \right)}{{G_{\max } }} = \frac{1}{{1 + e^{{\left( { - \frac{{V - V\raise.5ex\hbox{$\scriptstyle 1$}\kern-.1em/ \kern-.15em\lower.25ex\hbox{$\scriptstyle 2$} }}{k}} \right)}} }}$$

Inactivation Na^+^ currents were obtained using 20-ms test pulses to − 30 mV after a 500-ms pre-pulse to potentials ranging from − 120 to + 30 mV. Inactivation Ca^2+^ currents were obtained using 30-ms test pulses to + 10 mV after a 3000-ms pre-pulse to potentials ranging from − 40 to + 20 mV. The inactivation values were fitted to the following standard Boltzmann equation:

$$\frac{I\left( V \right)}{{I_{\max } }} = \frac{1}{{1 + e^{{\left( { - \frac{{V - V\raise.5ex\hbox{$\scriptstyle 1$}\kern-.1em/ \kern-.15em\lower.25ex\hbox{$\scriptstyle 2$} }}{k}} \right)}} }} + C$$

For Na^+^ recovery from inactivation, cells were depolarized to − 30 mV for 40 ms from a holding potential of − 100 mV to inactivate the Na^+^ channels. Test pulses were then applied at − 30 mV for 20 ms to measure current amplitudes, with intervals ranging from 0.06 to 4000 ms. The resulting curves were fitted with a double exponential equation. The time constants of fast Na^+^ and Ca^2+^ inactivation decay, were plotted as a function of voltage. The time constants were obtained using a simple exponential function. Window current was obtained thank to activation and inactivation parameters with the following formula:

$$Window\; current = \frac{1}{{1 + e^{{\left( { - \frac{{V\raise.5ex\hbox{$\scriptstyle 1$}\kern-.1em/ \kern-.15em\lower.25ex\hbox{$\scriptstyle 2$} activation - V}}{kactivation}} \right)}} }} \times \frac{1}{{1 + e^{{\left( { - \frac{{V - V\raise.5ex\hbox{$\scriptstyle 1$}\kern-.1em/ \kern-.15em\lower.25ex\hbox{$\scriptstyle 2$} inactivation}}{kinactivation}} \right)}} }} \times 100$$

For the current-clamp experiments, the patch pipettes (resistance 2–5 mΩ) were filled with a solution containing (in mmol/L) 10 NaCl, 122 KCl, 1 MgCl_2_, 1 EGTA, and 10 HEPES. The pH was adjusted to 7.3 with KOH. The bath solution was composed of (in mmol/L) 154 NaCl, 5.6 KCl, 2 CaCl_2_, 1 MgCl_2_, 8 D-glucose, and 10 HEPES. The pH was adjusted to 7.3 with NaOH. The APs were recorded at a 1-Hz stimulation frequency. The holding potential during the recordings was maintained at − 80 mV with the holding command mode of the amplifier or with a Cybercyte V10 dynamic clamp system (Suppl. Fig. S5) (Cytocybernetics, North TonaWanda, NY, USA). The duration of the stimulation pulse was 3 ms with 0.2- to 1.5-nA injected currents depending on the cell. The recorded resting membrane potential (RMP) was measured before establishing the holding potential of − 80 mV and before initiating the 1-Hz stimulation. To perform a classification of cell subtypes as ventricular-, atrial-, and nodal-like cardiomyocytes, we used an approach based on AP duration^57^. Cells exhibiting an action potential duration at 90% repolarization (APD_90_) exceeding 250 ms were automatically categorized as ventricular-like, while those with an APD_90_ shorter than 250 ms were classified as either atrial-like or nodal-like. To distinguish between atrial-like and nodal-like cells, we employed a criterion based on the difference between the APD_50_ and APD_20_ when stimulated at a frequency of 1 Hz. Cells displaying a difference less than 10 ms (APD_50_–APD_20_ ≤ 10 ms) were designated as nodal-like, whereas those surpassing this threshold were labeled as atrial-like. This particular criterion serves to emphasize the distinct AP profiles exhibited by nodal-like cells. We used a total of four independent differentiations to collect the electrophysiological data for the first cell line of iPSC-CMs, and three independent differentiations for the second cell line. These multiple independent differentiations allowed us to obtain robust and reliable electrophysiological measurements for our analysis.

### Optical mapping

vCMs and aCMs were dissociated at day 12 to 15, and cardiac monolayers were generated by seeding hiPSC-CMs (350,000 cells) on hESC-qualified Matrigel-coated (Cat# 07181, Corning) 13-mm TC coverslips (Cat# 83.1840.002, Sarstedt) in a 24-well plate. hiPSC-CM monolayers were cultured in STEMdiff™ Cardiomyocyte Maintenance Medium until used (30–40 days of maturation). For dual mapping, the monolayers were first stained with the Ca^2+^ indicator Rhod-2 AM (5 µmol/L, Cat# ab142780, Abcam) and incubated at 37 °C for 30 min, washed, loaded with the potentiometric dye RH 237 (15 µmol/L, Cat# S1109, ThermoFisher), and incubated for an additional 30 min. The cultures were then placed in an imaging solution containing (in mmol/L) 154 NaCl, 5.6 KCl, 2 CaCl_2_, 1 MgCl_2_, 8 D-glucose, and 10 HEPES, pH 7.3). The optical mapping system used to record AP and Ca^2+^ transients at 500 frames per second is an epifluorescence macroscope equipped with two CMOS N256 cameras (MiCAM03, Brainvision, SciMedia Ltd., USA), a 530 nm green LED light source (LEX2-LZ4-G), an imaging cube containing a collimator, a dichroic mirror (560 nm), and a bandpass excitation filter (50 mm, BrightLine^®^, Semrock). A second imaging cube was used as a beam splitter and contained a 662-nm dichroic mirror, two emission filters (572/15 nm, BrightLine^®^, Semrock), a longpass filter (715 nm, Andover Corporation), and a lens system with a maximum aperture of f/1.4 for RH 237 and Rhod-2 imaging.

The hiPSC-CMs were kept at 37 °C using a controlled heating plate (Multi Channel Systems, Reutlingen, Germany) and were electrically paced by two platinum/iridium electrodes positioned at the lower edge of the monolayers. A stimulus generator (STG4002, Multichannel Systems) was used to deliver 10-ms square bipolar pulses at a frequency of 1 Hz. Before adding blebbistatin (10 µmol/L, Cat# 72402, STEMCELL Technologies) to the imaging solution to prevent contraction artifacts, spontaneous electrical activity was recorded. Thereafter, all monolayers successfully paced at 1 Hz were analyzed. Raw optical signals were then processed, and were analyzed with Brainvision Workbench software (Brainvision, SciMedia) to extract the electrophysiological measurements, calculate the conduction velocities, and generate the activation maps.

### Statistical analysis

All statistical analyses were performed using PRISM10 software (GraphPad, CA, USA). The normality of the distribution was determined using the D'Agostino-Pearson normality test. The data are expressed as median ± quartiles (25% and 75%) with min to max values or as mean ± SEM (standard error of the mean). For the electrophysiological data, a replicate (n) represented the number of cells recorded. For the qPCR and Western blot data, a replicate (n) represented an RNA or protein extract from a single independent differentiation. For the optical mapping data, a replicate (n) corresponded to a hiPSC-CM monolayer in a 24-well plate. For two independent variables, such as the normalized intensity/voltage relationships (I/V) and the time constants of fast inactivation decay, a two-way ANOVA with a Šídák multiple comparisons test was used. Otherwise, a two-tailed unpaired Student’s t-test was used to compare aCM and vCM conditions. When three groups were compared (Fig. 5A), statistical significance was determined by one-way ANOVA with Tukey’s post hoc test. When the replicate (n) was too small (Suppl. Fig. S1A), a nonparametric Mann–Whitney test was performed. The Brubbs’ test, available on the GraphPad website, was performed to identify and exclude outliers that significantly deviated from the overall trend. All the statistical tests were performed using a 95% confidence interval, and the differences were considered significant beyond the 0.05% risk threshold (*p < 0.05, **p < 0.01, ***p < 0.001, and ****p < 0.0001).

### Supplementary Information

Supplementary Information.

## Data Availability

The raw data supporting the conclusion of this article will be made available by the authors, without undue reservation.
