# Supplementary material for: Biophysical properties of NaV1.5 channels from atrial-like and ventricular-like cardiomyocytes derived from human induced pluripotent stem cells
Source: Sci Rep. 2023 Nov 24;13:20685. doi: 10.1038/s41598-023-47310-6 (PMC10673932; doi:10.1038/s41598-023-47310-6)
Supplement: Supplementary file 1 — Supplementary Information. [file 41598_2023_47310_MOESM1_ESM.docx]

Supplementary Material

Biophysical properties of Na_V_1.5 channels from atrial-like and ventricular-like cardiomyocytes derived from human induced pluripotent stem cells

**Charles-Albert Chapotte-Baldacci^1,2^** **^†^, Marion Pierre^1,2^** **^†^, Mohammed Djemai^1,2^, Valérie Pouliot^2^ and Mohamed Chahine^1,2*^**

^1^Department of Medicine, Faculty of Medicine, Université Laval, Quebec City, Quebec, Canada

^2^CERVO Brain Research Centre, Quebec City, Quebec, Canada

^†^Equal contribution and first authorship: These authors contributed equally to this work and share first authorship

**^*^Correspondence:** Mohamed Chahine, Ph.D. Mohamed.Chahine@phc.ulaval.ca

# Supplementary Figures and Tables

## Supplementary Figures


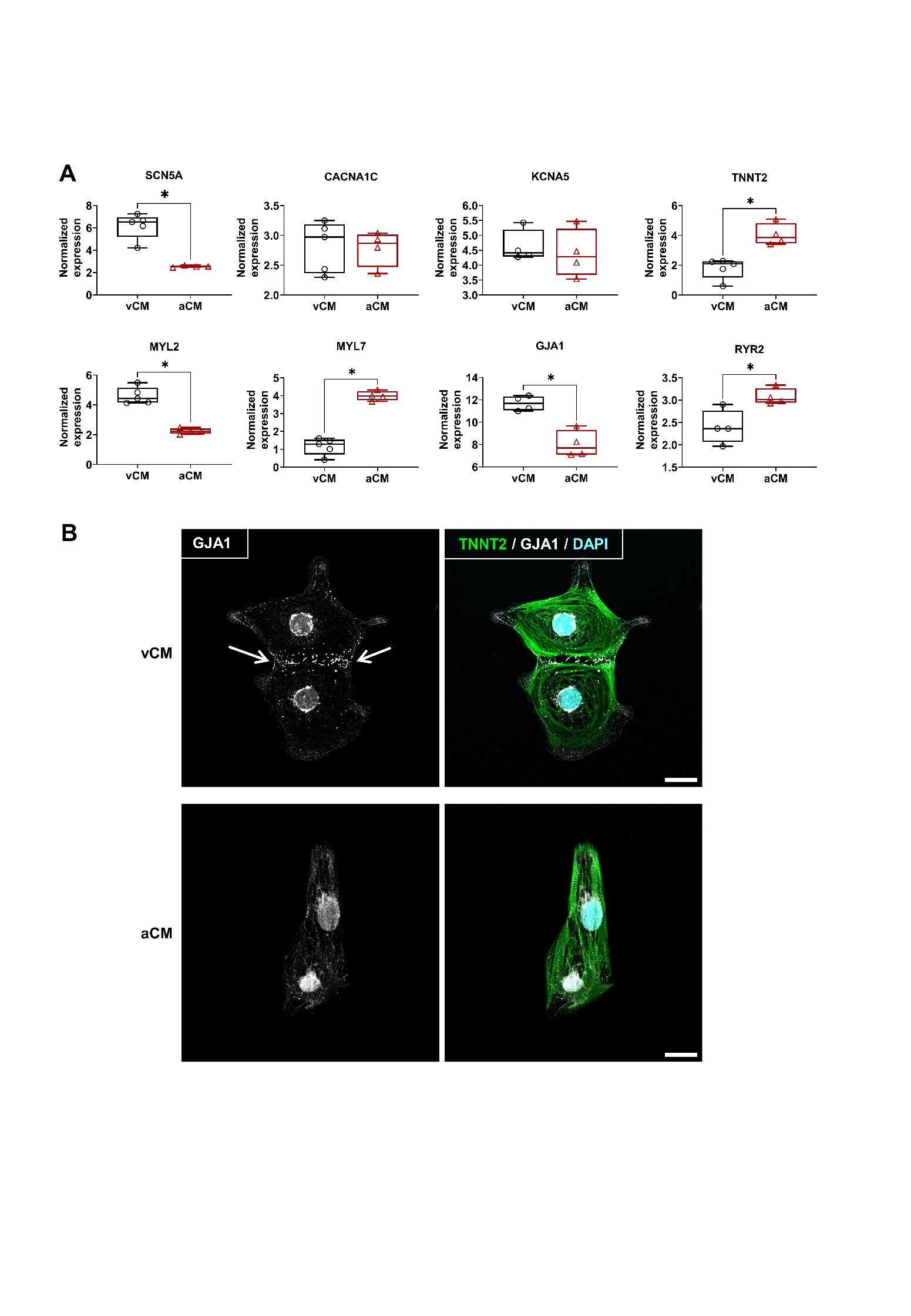


**Supplementary Figure S1. Protein expression in vCMs and aCMs. A:** Quantification of normalized protein expression obtained by Western-Blot. Each protein is normalized to the total protein of its lane. These quantifications are linked to Figure 1C. **B:** Confocal images magnified on GJA1 protein localization in vCMs and aCMs. Arrows showed the line of gap junction between adjacent cells (scale bar: 20 µm).


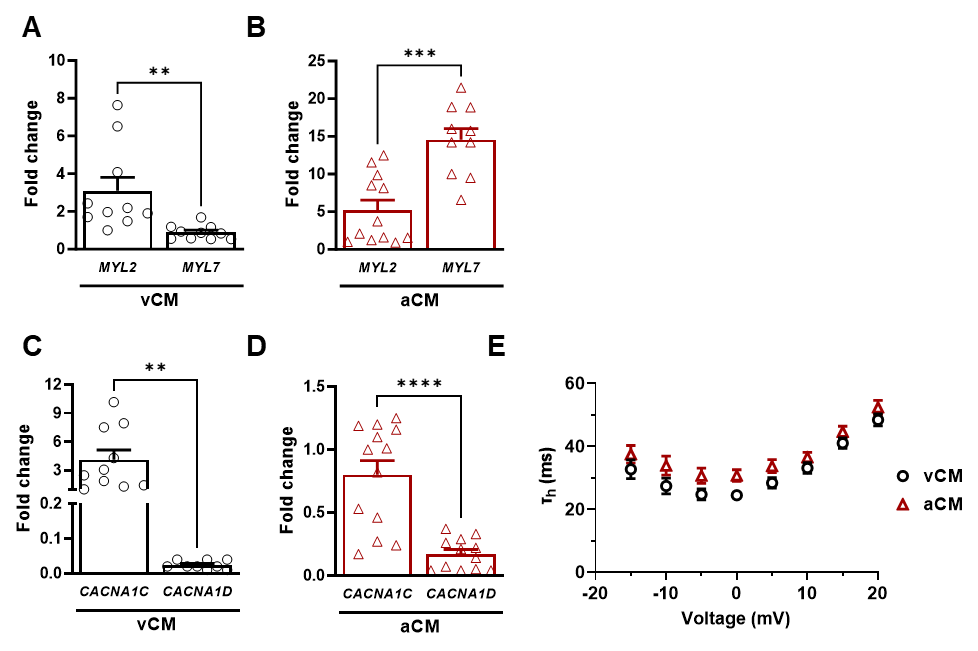
**Supplementary Figure S2. Additional information on *MYL2/MYL7* and *CACNA1C/CACNA1D* mRNA expressions and L-type VGCC biophysical properties in vCMs and aCMs.** **A:** qPCR analysis of *MYL2* and *MYL7* mRNA expression in vCMs. *MYL2* mRNA in vCMs served as reference. **B:** qPCR analysis of *MYL2* and *MYL7* mRNA expression in aCMs. *MYL2* mRNA in vCMs served as reference. **C:** qPCR analysis of *CACNA1C* and *CACNA1D* mRNA expression in vCMs. *CACNA1C* mRNA in vCMs served as reference. **D:** qPCR analysis of *CACNA1C* and *CACNA1D* mRNA expression in aCMs. *CACNA1C* mRNA in aCMs served as reference. **E:** The time constants of inactivation decay plotted as a function of voltage of L-type VGCCs.


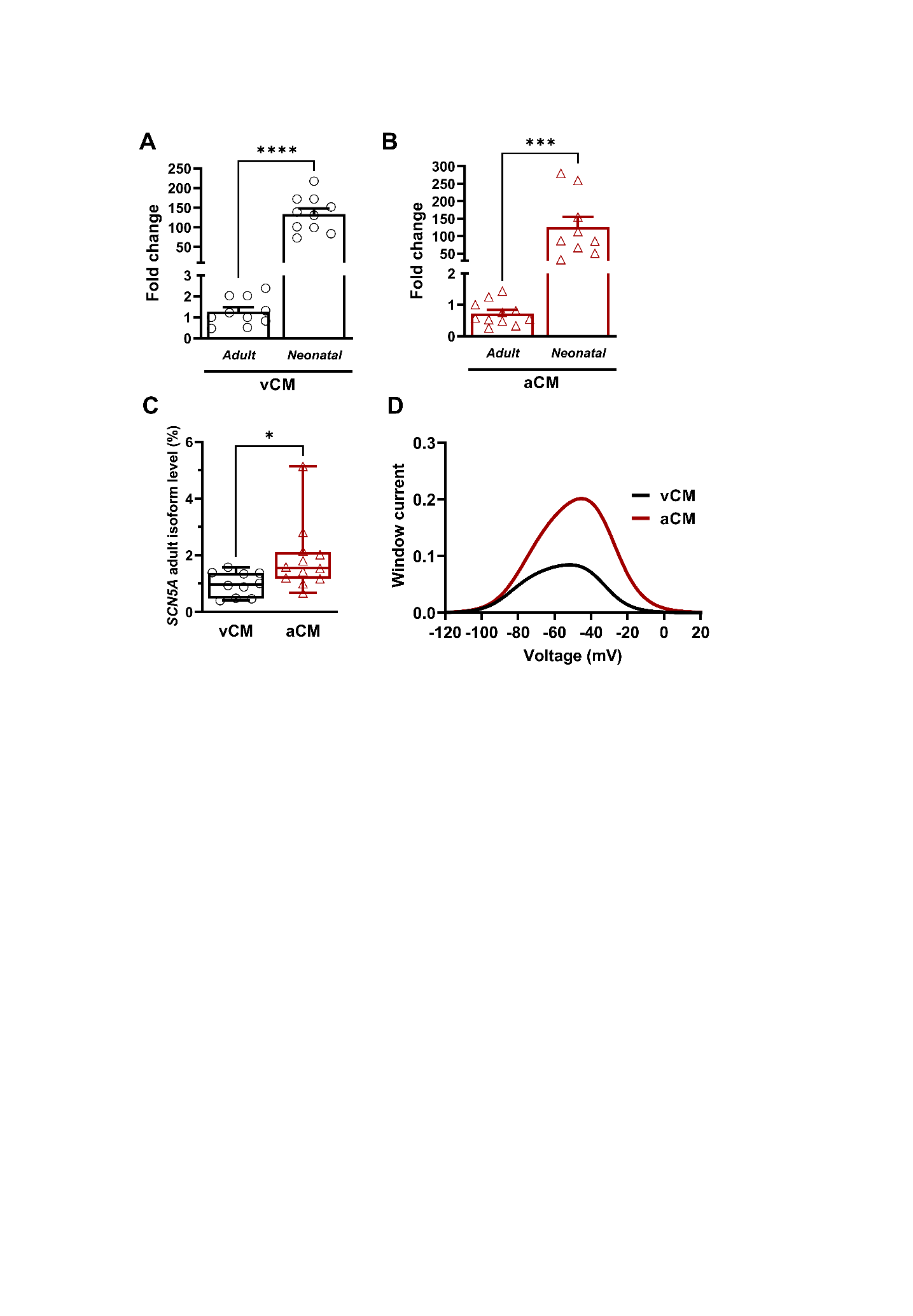
**Supplementary Figure S3.**  **Additional information about Na_V_1.5 biophysical properties and *SCN5A* adult/neonatal isoform expression. A:** qPCR analysis of *SCN5A* exon 6b (adult) and *SCN5A* exon 6a (neonatal) mRNA expression in vCMs. *SCN5A* exon 6b (adult) mRNA in vCMs served as reference. **B:** qPCR analysis of *SCN5A* exon 6b (adult) and *SCN5A* exon 6a (neonatal) mRNA expression in aCMs. *SCN5A* exon 6b (adult) mRNA in aCMs served as reference. **C:** *SCN5A* adult isoform expression in vCMs and aCMs. Percentage of adult isoform obtained by the ratio: isoform adult (exon 6b) / isoform neonatal (exon 6a) x 100. **D:** Window current of Na_V_1.5 channels in vCMs and aCMs. The window current represents the overlap between activation and inactivation.


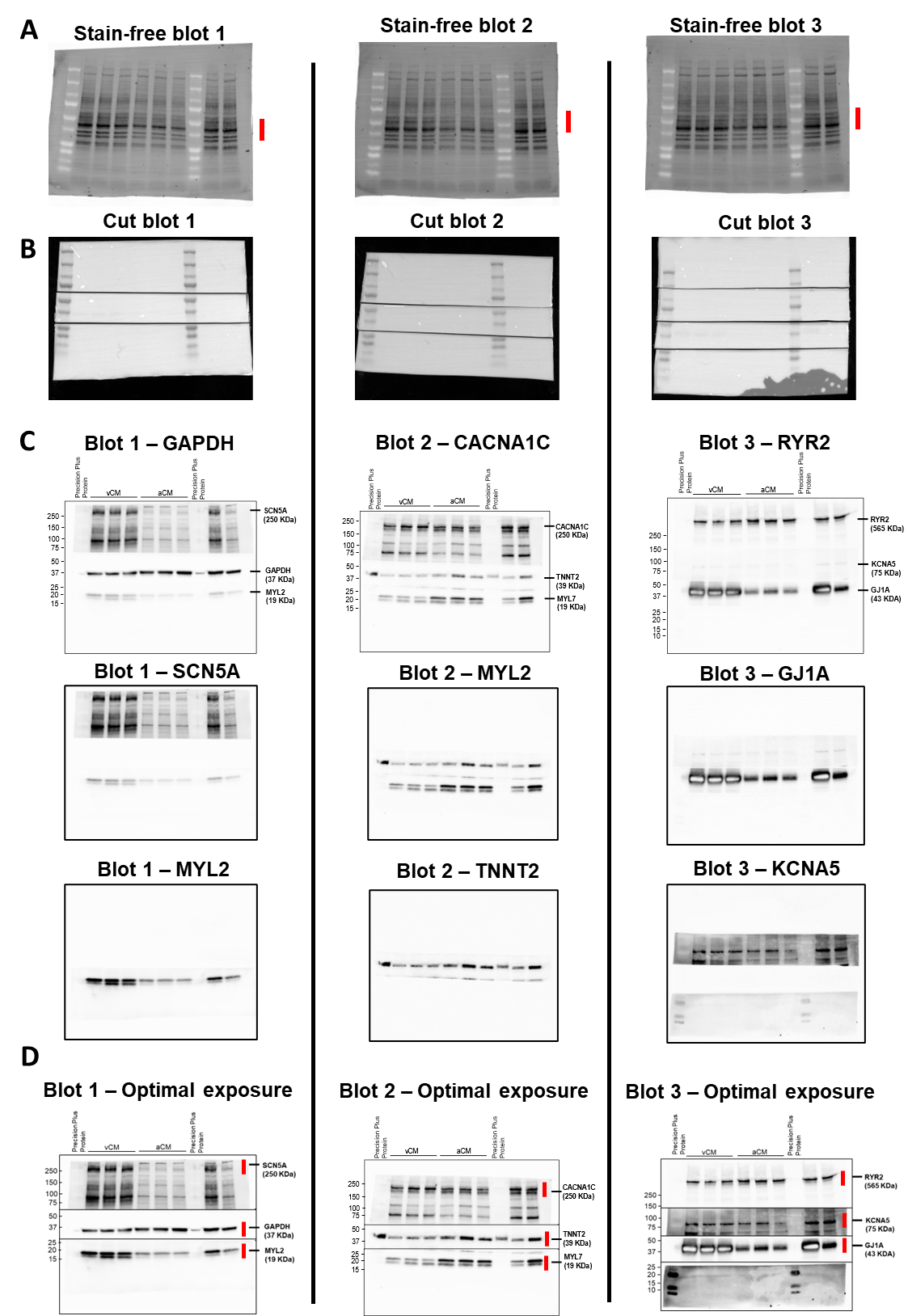


**Supplementary Figure S4. Immunoblotting details. A:** Pictures of stain-free blots showing total proteins after protein transfers. **B:** Colorimetric pictures of protein ladder. Each blot was cut into 3 or 4 sections prior to blotting. Blots were cut using a razor blade. Each section was incubated with a antibodies and blot sections were replaced before image acquisitions. Blots were cut prior to blotting with primary antibodies because several of selected primary antibodies belong to same species. **C:** Native western-blot pictures labelled with antibodies targeting specific proteins. All blots were acquired using a BioRad ChemiDoc MP imaging system using the auto-exposure function, which enables unsaturated image acquirement. All blots were exposed at the same time, then individually removed when near saturation to properly auto expose. **D:** Optimal exposure picture selected for each protein. Area of the blot sections has been delineated with black lines. Blots in the Figure 1 depict the best exposure time for each protein. Red lines indicate the area of the cropped blots found in the Figure 1. Blot 1 refers to the top panel of the Figure 1C, Blot 2 refers to the middle panel of the figure 1C and Blot 3 refers to the bottom panel of the figure 1C.


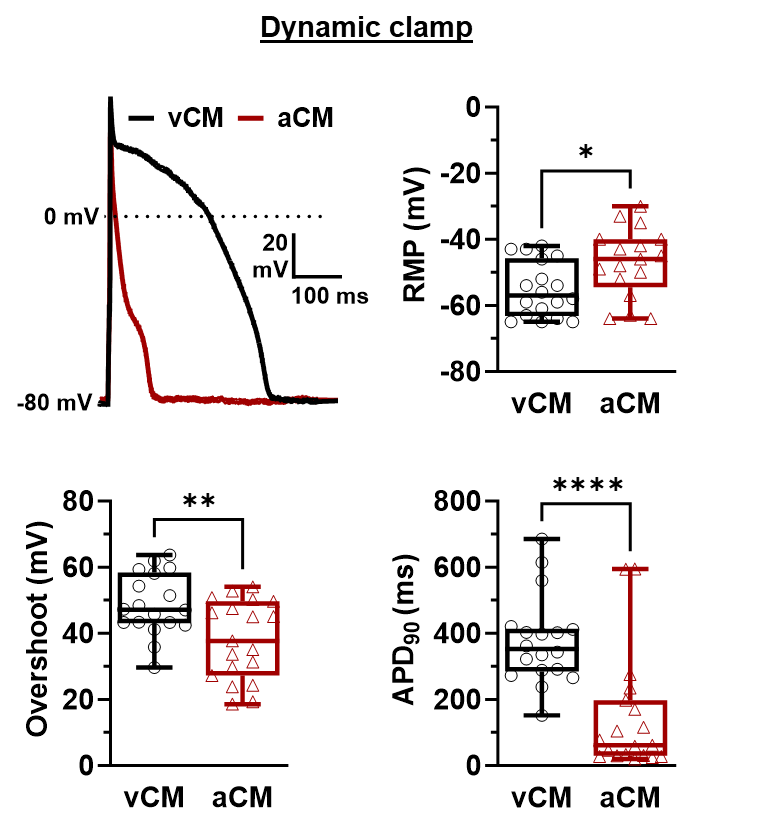


**Supplementary Figure S5. AP recordings using a dynamic-clamp system.** Representative AP traces recorded at 1 Hz (Top left), resting membrane potential (Top right), overshoot (Bottom left), and the action potential duration at 90% of repolarization (Bottom right) recorded using a commercially available dynamic-clamp system. The experiments were conducted using a Cybercyte V10 dynamic clamp system (Cytocybernetics, North TonaWanda, NY, USA). This allowed us to mimic the dynamics of the I_K1_ current, which plays a significant role at the end of the repolarization phase, helping to maintain the membrane potential close to -80 mV. The experiments were carried out on hiPSC-CMs stimulated at 1 Hz to normalize the beating rate and enable further analysis.


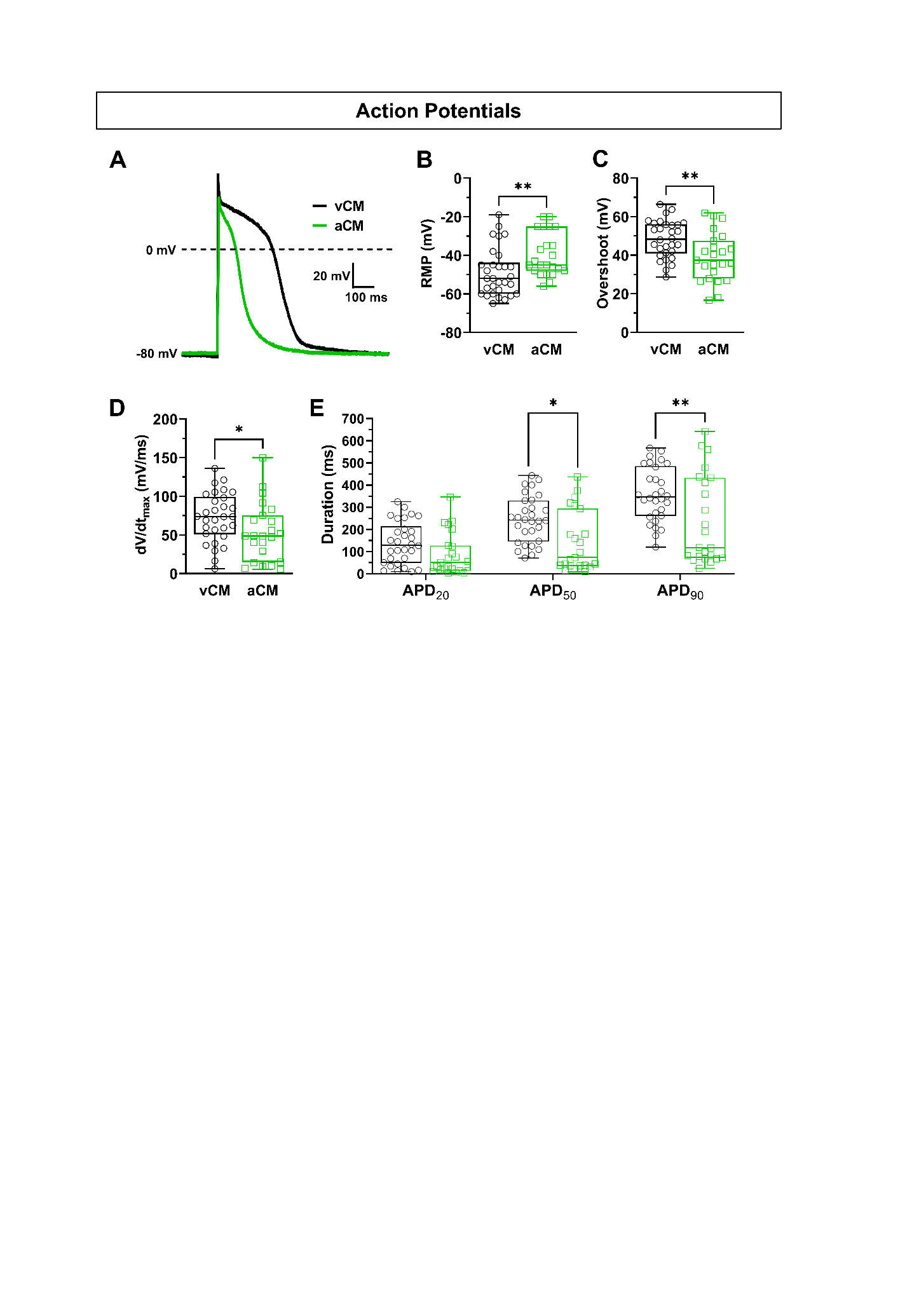
**Supplementary Figure S6. Characterization of cardiac action potentials of a second cell line of vCMs and aCMs. A:** Superposed APs recorded in current-clamp mode at 1 Hz. The dashed line represents 0 mV. **B, C, D, E:** Box and whiskers summarizing the resting potential membrane (B), the overshoot (C), the dV/dt_max_ (D) and the APD at 20, 50 and 90% of repolarization (E).


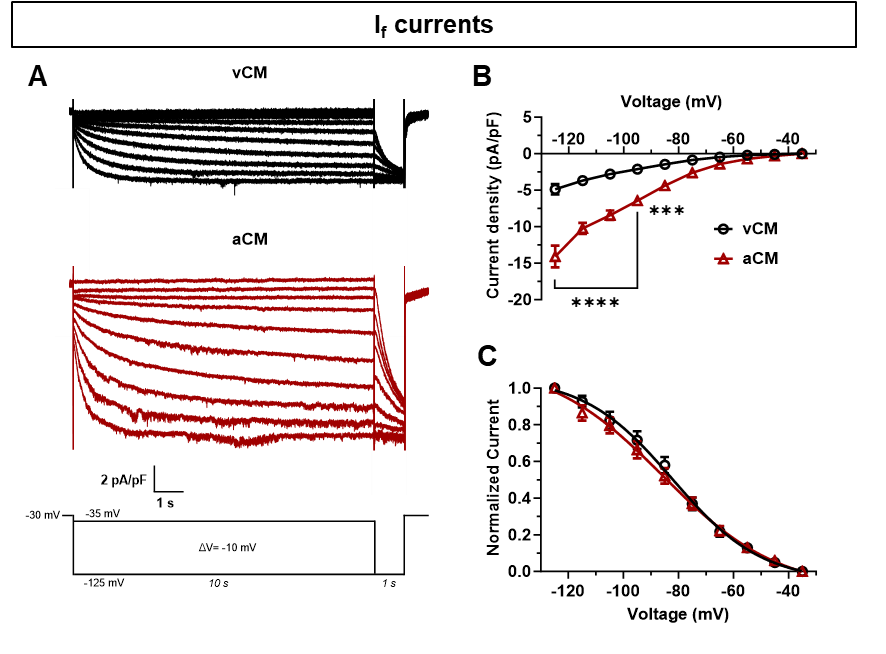
**Supplementary Figure S7. Characterization of I_f_ current in vCMs and aCMs. A:** Representative I_f_ current density in vCMs and aCMs. The patch-clamp protocol used is represented below. **B:** Normalized intensity/voltage relationships (I/V) obtained from currents at the end of the voltage steps. I_f_ current density was measured by normalizing current amplitudes to membrane capacitance. **C:** Steady state of activation determined from tail currents.


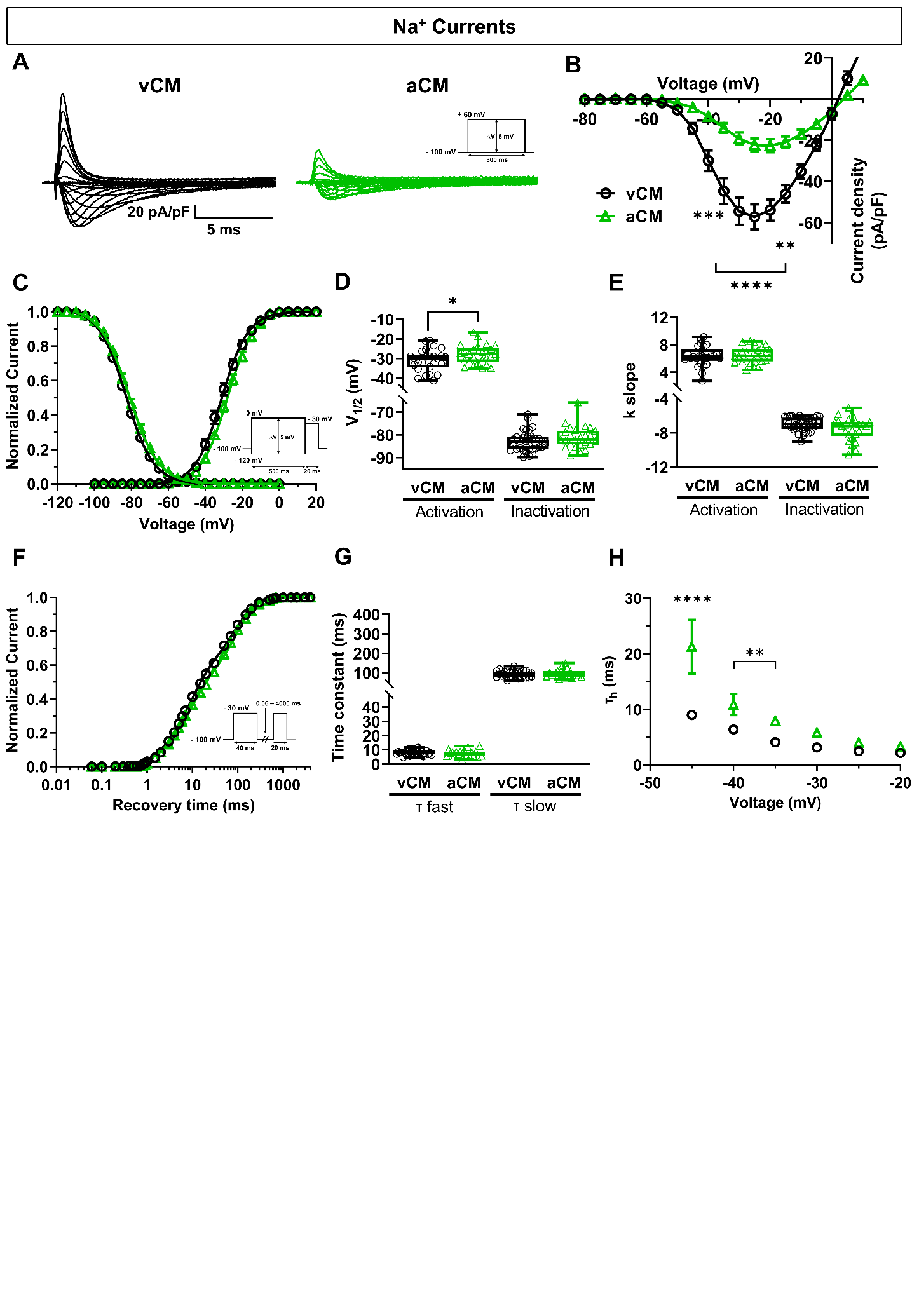


**Supplementary Figure S8. Biophysical properties of Na^+^ channels in a second cell line of vCMs and aCMs.** **A:** Representative Na^+^ current densities recorded in voltage-clamp mode. The dashed line represents zero current. **B:** Normalized intensity/voltage relationships (I/V). **C:** Steady-state activation and inactivation of Na^+^ currents. **D, E:** Box and whiskers summarizing the half-activation and half-inactivation potentials (D) and the k slope (E). **F:** Recovery from inactivation. **G:** Box and whiskers summarizing the recovery time constants. **H:** The time constants of fast inactivation decay plotted as a function of voltage.

## Supplementary Tables

**Supplementary Table S1. Parameters of electrical activity and calcium handling.**

| Parameters | Conditions | | | | | |
| --- | --- | --- | --- | --- | --- | --- |
|  | vCMs | | aCMs | | | *p* value |
|  | (Mean ± SEM | n) | (Mean ± SEM | n) | |  |
| Action potentials (APs) in isolated hiPSC-CMs | | | | | | |
| Membrane properties |  | 26 |  | 29 | |  |
| Resting membrane potential RMP (mV) | -66.04 ± 0.59 |  | -44.38 ± 2.08 | |  | ∗∗∗∗ |
| Threshold of depolarization (nA) | 0.47 ± 0.03 |  | 0.38 ± 0.02 | |  | ∗ |
| AP parameters |  | 26 |  | 29 | |  |
| Overshoot (mV) | 55.39 ± 1.66 |  | 43.13 ± 2.21 | |  | ∗∗∗∗ |
| Max. upstroke velocity dV/dt_max_ (mV/ms) | 87.82 ± 6.31 |  | 68.28 ± 5.49 | |  | ∗ |
| AP durations |  | 26 |  | 29 | |  |
| At 20% of repolarization APD_20_ (ms) | 154.00 ± 19.14 |  | 18.75 ± 3.42 | |  | ∗∗∗∗ |
| At 50% of repolarization APD_50_ (ms) | 325.80 ± 18.23 |  | 47.21 ± 5.46 | |  | ∗∗∗∗ |
| At 90% of repolarization APD_90_ (ms) | 419.80 ± 19.01 |  | 132.00 ± 13.79 | |  | ∗∗∗∗ |
| Activation map |  | 10 |  | 6 | |  |
| Conduction velocity CV (cm/s) | 8.78 ± 0.44 |  | 1.90 ± 0.19 | |  | ∗∗∗∗ |
| Spontaneous beating frequency (Hz) | 0.68 ± 0.06 |  | 1.42 ± 0.17 | |  | ∗∗∗ |
| OAP parameters |  | 10 |  | 6 | |  |
| Max. upstroke velocity dV/dt_max_ (U/s) | 3509 ± 170 |  | 2898 ± 661 | |  | *ns* |
| Peak amplitude (dF/F_0_, A.U.: arbitrary unit) | 0.84 ± 0.01 |  | 0.80 ± 0.03 | |  | *ns* |
| Time constant decay τ (ms) | 287.20 ± 13.18 |  | 90.62 ± 13.44 | |  | ∗∗∗∗ |
| OAP durations |  | 10 |  | 6 | |  |
| At 20% of repolarization APD_20_ (ms) | 231.72 ± 25.62 |  | 89.75 ± 23.80 | |  | ∗∗∗∗ |
| At 50% of repolarization APD_50_ (ms) | 333.41 ± 43.07 |  | 123.42 ± 33.18 | |  | ∗∗∗∗ |
| At 80% of repolarization APD_80_ (ms) | 395.16 ± 54.36 |  | 162.31 ± 41.79 | |  | ∗∗∗∗ |
| Activation map |  | 10 |  | 6 | |  |
| Ca^2+^ propagation velocity CaPV (cm/s) | 8.67 ± 0.77 |  | 2.26 ± 0.47 |  | | ∗∗∗∗ |
| CT parameters |  | 10 |  | 6 | |  |
| Half rise to peak time (ms) | 265.4 ± 6.0 |  | 51.1 ± 3.6 | |  | ∗∗∗∗ |
| Peak amplitude (dF/F_0_) | 0.96 ± 0.01 |  | 0.91 ± 0.01 | |  | ∗∗∗ |
| Time constant decay τ (ms) | 411.8 ± 4.3 |  | 197.1 ± 10.4 | |  | ∗∗∗∗ |
| CT durations |  | 10 |  | 6 | |  |
| At 50% of CT TD_50_ (ms) | 606.13 ± 22.59 |  | 200.51 ± 28.52 | |  | ∗∗∗∗ |
| At 80% of CT TD_80_ (ms) | 785.85 ± 15.73 |  | 350.26 ± 30.05 | |  | ∗∗∗∗ |

Data are represented in mean ± SEM and the replicate (n) represents the number of recorded cells. The significance was determined using the unpaired t-test. *ns*: no significant, ∗*p*<0.05, ∗∗*p*<0.01, ∗∗∗*p*<0.001 and ∗∗∗∗*p*<0.0001.

**Supplementary Table S2. Parameters of Na^+^ and Ca^2+^ biophysical properties.**

| Parameters | Conditions | | | | |
| --- | --- | --- | --- | --- | --- |
|  | vCMs | | aCMs | | *p* value |
|  | (Mean ± SEM | n) | (Mean ± SEM | n) |  |
| Na^+^ channel properties | | | | | |
| Na^+^ currents |  | 30 |  | 30 |  |
| Membrane capacitance Cm (pF) | 24.88 ± 1.62 |  | 20.09 ± 1.14 |  | ∗ |
| Current density at -30 mV (pA/pF) | -72.60 ± 8.16 |  | -26.59 ± 3.03 |  | ∗∗∗∗ |
| Conductance Gmax (pS) | 91.51 ± 10.19 |  | 37.69 ± 4.81 |  | ∗∗∗∗ |
| Steady-state activation |  | 30 |  | 30 |  |
| Half potential V_1/2_ (mV) | -33.86 ± 0.98 |  | -29.09 ± 1.03 |  | ∗∗∗ |
| K slope factor | 6.65 ± 0.29 |  | 7.25 ± 0.23 |  | *ns* |
| Steady-state inactivation |  | 30 |  | 30 |  |
| Half potential V_1/2_ (mV) | -86.15 ± 0.65 |  | -80.69 ± 0.77 |  | ∗∗∗∗ |
| K slope factor | -6.91 ± 0.19 |  | -7.88 ± 0.23 |  | ∗ |
| Recovery from inactivation |  | 30 |  | 30 |  |
| Time constant τ fast (ms) | 11.22 ± 1.38 |  | 7.65 ± 0.56 |  | *ns* |
| Time constant τ slow (ms) | 112.2 ± 8.42 |  | 107.3 ± 4.32 |  | *ns* |
| Ca^2+^ currents |  | 20 |  | 23 |  |
| Current density at 5 mV (pA/pF) | -12.77 ± 1.29 |  | -8.63 ± 0.65 |  | ∗∗∗∗ |
| Conductance Gmax (pS) | 11.18 ± 1.42 |  | 6.32 ± 0.59 |  | ∗∗ |
| Steady-state activation |  | 20 |  | 23 |  |
| Half potential V_1/2_ (mV) | -7.18 ± 0.72 |  | -6.40 ± 0.56 |  | *ns* |
| K slope factor | 5.54 ± 0.25 |  | 6.65 ± 0.27 |  | ∗∗ |
| Steady-state inactivation |  | 10 |  | 18 |  |
| Half potential V_1/2_ (mV) | -33.28 ± 1.05 |  | -33.77 ± 1.06 |  | *ns* |
| K slope factor | -4.79 ± 0.20 |  | -5.60 ± 0.31 |  | *ns* |

Data are represented in mean ± SEM and the replicate (n) represents the number of recorded cells. The significance was determined using the two-way ANOVA with Sidak multiple comparison test or the unpaired t-test. *ns*: no significant, ∗p<0.05, ∗∗p<0.01, ∗∗∗p<0.001 and ∗∗∗∗p<0.0001.

| **Supplementary Table S3. Human gene-specific primers for qPCR** | | | | | |
| --- | --- | --- | --- | --- | --- |
| **Gene** | **Primers (5'-3')** | | | **Tm (°C)** | |
|  | **Forward** | **Reverse** | |  |  |
| *PPIA* | TTCATCTGCACTGCCAAGAC | | TCGAGTTGTCCACAGTCAGC | | 61 |
| *RPL22* | CCATGGCTCCTGTGAAAAA | | TCACGGTGATCTTGCTCT | | 61 |
| *SCN1B* | AGGATCTGTCTATCTTCATCAC | | ATGTCTCTGTTGGCTTTGTC | | 60 |
| *SCN2B* | TCTGAGGAGATGTTCCTCCA | | CATGATGTAGCAGTTGTAAATCCC | | 58 |
| *SCN3B* | ACATCCTTCTGGTCTTCCTC | | ACACTGCTCCTGTTCTATTCC | | 60 |
| *SCN4B* | CGCATTCAAGATTCTCATAGAGG | | AACGACTTGGAGGAAGATGG | | 60 |
| *CACNA1C* | GTCCAGCACACCTCCTTCAG | | AGCCCCATAAGCAGTCATCTTC | | 58 |
| *CACNA1D* | GCTGTTTGGCGGCAAGTTTA | | CTTCGCCTGTCAGGATCTGG | | 58 |
| *GJA1* | TGAGCAGTCTGCCTTTCGTT | | CCAGAAGCGCACATGAGAGA | | 58 |
| *MYL2* | CCTTTCCACCATGGCACCT | | AAGCCATCCCTGTTCTGGTC | | 61 |
| *MYL7* | ATCTGCAAGGCAGACCTGAG | | TGAAGTTGATGGGGCCCTTG | | 61 |
| *SCN5A* (adult) | GCATACACAACTGAATTTGTGG | | GTCTTCAGCCCTGAAATGAC | | 61 |
| *SCN5A* (neonatal) | AGTATGTCGAGTACACCTTCAC | | CTGAAAGTTCGAAGAGCCGAC | | 61 |
| *SCN5A* (exon 25) | ATGAAGAGCAGCCTCAGTGG | | CCAATAAAGAGGTTCAGGGTGA | | 58 |

Tm: melting temperature, *PPIA*: Peptidylprolyl Isomerase A, *RPL22*: Ribosomal Protein L22, *SCN1B*: Sodium Voltage-Gated Channel Beta Subunit 1, *SCN2B*: Sodium Voltage-Gated Channel Beta Subunit 2, *SCN3B*: Sodium Voltage-Gated Channel Beta Subunit 3, *SCN4B*: Sodium Voltage-Gated Channel Beta Subunit 4, *CACNA1C*: Calcium Voltage-Gated Channel Subunit Alpha1 C, *CACNA1D*: Calcium Voltage-Gated Channel Subunit Alpha1 D, *GJA1*: Gap Junction Protein Alpha 1, *MYL2*: Myosin Light Chain 2, *MYL7*: Myosin Light Chain 7, *SCN5A*: Sodium Voltage-Gated Channel Alpha Subunit 5.
